# Physical activity phenotyping with activity bigrams, and their association with BMI

**DOI:** 10.1093/ije/dyx093

**Published:** 2017-06-29

**Authors:** Louise AC Millard, Kate Tilling, Debbie A Lawlor, Peter A Flach, Tom R Gaunt

**Affiliations:** dyx093-1 MRC Integrative Epidemiology Unit (IEU),; dyx093-2 School of Social and Community Medicine and; dyx093-3 Intelligent Systems Laboratory, University of Bristol, Bristol, UK

**Keywords:** Physical activity, bigrams, body mass index, ALSPAC

## Abstract

**Background:**

Analysis of physical activity usually focuses on a small number of summary statistics derived from accelerometer recordings: average counts per minute and the proportion of time spent in moderate-vigorous physical activity or in sedentary behaviour. We show how bigrams, a concept from the field of text mining, can be used to describe how a person’s activity levels change across (brief) time points. These variables can, for instance, differentiate between two people spending the same time in moderate activity, where one person often stays in moderate activity from one moment to the next and the other does not.

**Methods:**

We use data on 4810 participants of the Avon Longitudinal Study of Parents and Children (ALSPAC). We generate a profile of bigram frequencies for each participant and test the association of each frequency with body mass index (BMI), as an exemplar.

**Results:**

We found several associations between changes in bigram frequencies and BMI. For instance, a one standard deviation decrease in the number of adjacent minutes in sedentary then moderate activity (or vice versa), with a corresponding increase in the number of adjacent minutes in moderate then vigorous activity (or vice versa), was associated with a 2.36 kg/m^2^ lower BMI [95% confidence interval (CI): −3.47, −1.26], after accounting for the time spent in sedentary, low, moderate and vigorous activity.

**Conclusions:**

Activity bigrams are novel variables that capture how a person’s activity changes from one moment to the next. These variables can be used to investigate how sequential activity patterns associate with other traits.

**Key Messages**

- Epidemiologists typically use only a small number of variables to analyse the association of physical activity with other traits, such as the average counts per minute and the proportion of time spent in moderate-vigorous physical activity or being sedentary.
- We demonstrate how activity bigrams can be used as a set of interpretable variables describing how a person’s activity levels change from one moment to the next.
- Testing the association of activity bigrams with exposures or outcomes can help us gain further understanding of how physical activity is associated with other traits; with further research they might provide evidence for more refined public health advice.

## Introduction

Physical activity—defined as any bodily movement that results in energy expenditure—is associated with many diseases, such as diabetes^1^ and coronary heart disease.^2^ Research increasingly uses objective measures of physical activity recorded using accelerometers, rather than self-report via questionnaires that are affected by reporting bias and measurement error.^3^ Cohort participants wear an accelerometer that measures accelerations at time intervals typically ranging from 0.01 s^4^ to 1 min.^5^^,^^6^ These high-resolution time-series data potentially contain much valuable information about a person’s activity. To date, however, only a small number of variables derived from accelerometer recordings have been used: average counts per minute (mCPM), and the proportion of time spent in moderate-vigorous physical activity (MVPA) or sedentary behaviour (SB).^5^^,^^7–12^ These measures only include a fraction of the information contained in accelerometer sequences, and this may lead to bias and a loss of power when using these variables as measures of physical activity. As public health advice has, to date, been informed by research using these limited variables, it is possible that analyses using other aspects of this data would support more refined advice.

Recently, work has been conducted to generate other variables describing physical activity. Goldsmith *et al.* used functional data analyses to model diurnal physical activity profiles and test the association of these profiles with other traits.^13^ For example, they identified that during daytime hours, girls are less active than boys, but this difference is not present in the evening. Evenson *et al.* used latent class analysis to assign participants to groups based on their activity levels across a 1-week period, to identify common weekly patterns of activity.^14^ Their analyses using MVPA identified an interesting set of four groups—two lower-activity groups with stable MVPA across the week, and two higher-activity groups, one most active between Monday and Thursday and the other most active between Friday and Sunday. Augustin *et al.* used a histogram of activity counts as a functional summary of activity,^15^ which is beneficial because it does not assume that the association of activity on a second trait across activity levels is linear, and also allows this assumption to be tested. Their analysis with fat mass found a non-linear association across activity intensities.

One key aspect of accelerometer sequences not captured by mCPM and time spent in different intensities of activity from sedentary to MVPA, or the more recent methods described above, is variability in a person’s activity levels from one moment to the next. For instance, two people may have the same mCPM and also the same total time spent in MVPA, but the first person may stay at the vigorous activity level for one continuous period, whereas the second enters into the vigorous activity state for more frequent, shorter bouts. It is increasingly recognized that variability of a trait about the mean level can have important associations with exposures and outcomes, independently of the mean level (e.g. variation of systolic blood pressure).^16–18^

At present, most physical activity guidelines recommend accumulating at least 150 min of moderate intensity activity or 75 min of vigorous intensity activity a week,^19^^,^^20^ with no advice on possible benefits of time-varying intensities. There is increasing interest in the possible health benefits of undertaking repeat short bursts of high-intensity activity, referred to as high-intensity interval training (HIIT).^21–23^ HIIT research assesses the benefits of short periods of very high-intensity activity, but there are many other sequential activity patterns that might also be beneficial (or detrimental) to a person’s health. However, methods for assessing the association of a sequence of exposures (rather than the mean level) with an outcome are not widely used.

Sequential data, like those from accelerometers, occur in many settings. The field of text mining seeks to learn models to make predictions from the sequence of words in a document.^24^ A common approach is to treat each document as an unordered collection of words, each called a unigram, and to use the frequency of each word in the document as a variable in analyses. The set of words and associated frequencies is known as a bag of words. This is equivalent to the way epidemiologists treat accelerometer data, after the sequence is first categorized into sedentary, low and moderate/vigorous activity. The accelerometer sequence is treated as an unordered collection of these activity categories and the variables MVPA and SB denote the frequency (or proportion) of each category in the sequence. Hence we can view these activity categories as activity unigrams*,* and the set of activity unigrams with the associated frequencies as a bag of activity unigrams. A unigram is a sequence of length one, and this can be generalized to *n*-grams—sequences of length *n*—and bags of *n*-grams (see Supplementary material Section S1 for examples, available at *IJE* online). This provides opportunities to extend representations of physical activity beyond activity unigrams.

In this paper we use 2-grams, referred to as bigrams, to represent sequential patterns in a person’s accelerometer sequence. This is useful as we can then ask, for instance, how often is a person in the moderate state at time *t* and the vigorous state at time *t + 1*? Activity bigrams can be used to examine how changes in activity from one moment to the next associate with other traits. We demonstrate our novel approach with body mass index (BMI) as an exemplar.

## Methods

### Participants

We used data on participants in the Avon Longitudinal Study of Parents and Children (ALSPAC), a prospective population-based cohort. The ALSPAC study website contains details of all the data that are available through a fully searchable data dictionary: [http://www.bris.ac.uk/alspac/researchers/data-access/data-dictionary/]. The study methods are described in detail elsewhere.^25^ In brief, ALSPAC recruited 14 541 pregnant women resident in Avon, UK, with expected dates of delivery between 1 April 1991 and 31 December 1992 [http://www.alspac.bris.ac.uk]. These mothers and their children have been followed with regular assessments since this time. Ethical approval for the study was obtained from the ALSPAC Ethics and Law Committee and the local research ethics committees.

### Data collection

Physical activity was measured using the uni-axial Actigraph 7164 accelerometer that measures vertical accelerations. All children who attended the age 11 clinic were asked to wear an accelerometer on their waist for 7 days, taking it off while sleeping, showering, bathing or swimming. The devices were programmed to start recording at 05:00 a.m. the day after the clinic. The sum of activity counts (a measure of acceleration) over each 1-min epoch (interval) was recorded, giving a maximum sequence of 10 080 values for each participant. We refer to each 2-min interval in a person’s sequence as an epoch pair. The total number of epoch pairs in a sequence is equal to the length of the sequence minus one.

Weight and height were measured at the age 11 clinic, with the child in light clothing without shoes. BMI was calculated as weight in kilograms divided by height in metres squared. We consider the following potential confounders: child gender, exact age at age 11 clinic, parity, household social class, maternal education, maternal smoking during pregnancy and child ethnicity (details of how these were assessed are provided in Supplementary material Section S2, available at *IJE* online).

### Study sample

**Figure 1:**
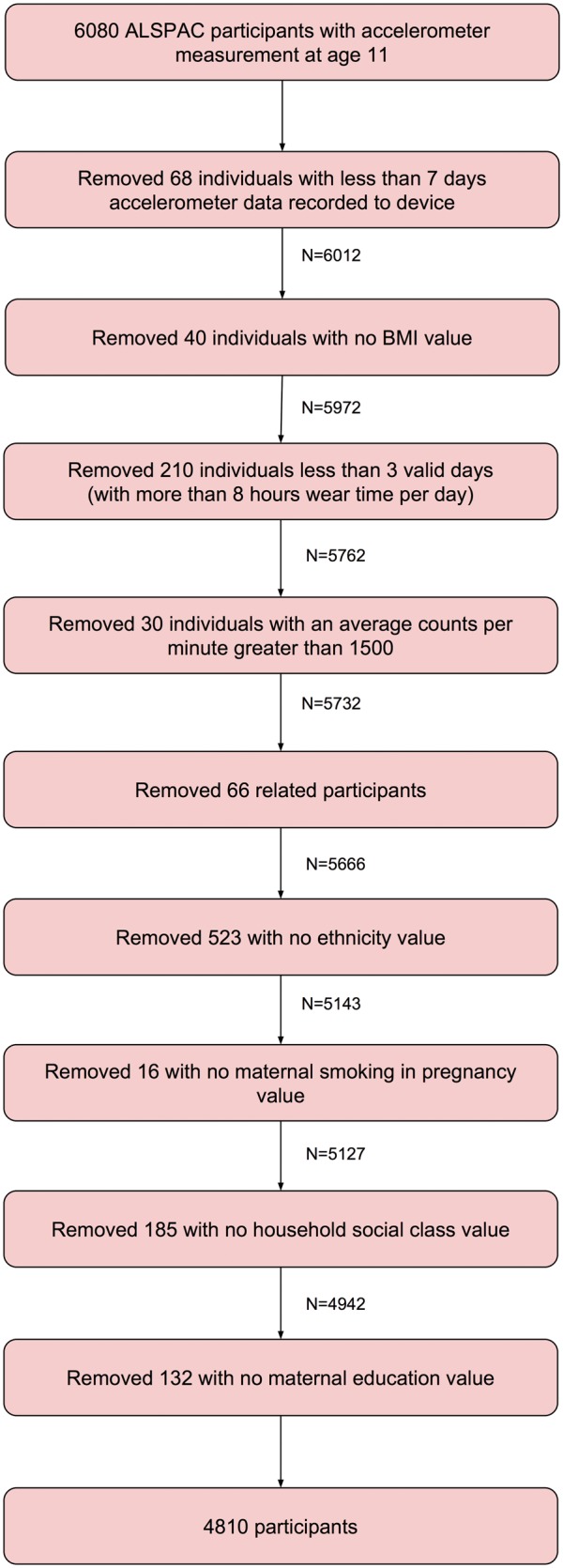
Participant flow diagram showing creation of our sample in ALSPAC.

Of the 6080 participants with accelerometer data, we excluded from analyses 68 participants who did not have 7 days of recorded accelerometer data. We further excluded 40 participants with no measure of BMI. We assumed that continuous sequences of zero activity counts of length greater than 60 epochs (1 hour) meant that the participant was not wearing their device, and treated such periods as missing accelerometer data.^26^ Accelerometer data were considered invalid if: (i) there were fewer than three valid days, where a valid day is defined as at least 8 h of wear time; or (ii) the average activity level per minute was greater than 1500, as this was deemed infeasible. We excluded 240 participants with invalid accelerometer data. We removed 66 participants who were siblings to other participants in this sample. We excluded a further 856 participants with no value for at least one confounding factor, giving a resultant sample size of 4810 participants. A participant flow diagram is given in Figure 1.

### Statistical analyses

The activity levels at each epoch of participant’s accelerometer sequences (excluding non-wear time) were categorized into four groups of activity intensities: sedentary, 0–100 activity counts per minute; low, 101–2019; moderate, 2020–5998; and vigorous, 5999+,^6^^,^^26^ denoted S, L, M and V, respectively. We refer to these as activity states, to distinguish from the continuous activity levels of the original accelerometer data.

#### Relating mean activity levels to outcomes

We use univariate linear regression (regress function in Matlab) to test the association of the mean activity levels per minute over the time where a participant wore the accelerometer (mCPM) and the standard deviation of these activity levels per minute (sdCPM), individually, with BMI. We test this association before and after adjustment for potential confounding factors, and also after mutual adjustment of mCPM and sdCPM.

Within 1 day there are a finite number of occurrences of activity states in total (the number of minutes = 1440), such that as the frequency of one activity state increases, this must be coupled with a decrease in frequency of at least one other activity state. This means that an increase in frequency of the moderate state may, for instance, be associated with lower BMI when the additional frequency comes from the sedentary state, but not when it comes from the vigorous state. We calculate the average number of minutes each participant spends in S, L, M and V activity states per day, denoted *S_d_*, *L_d_*, *M_d_* and *V_d_*, respectively. We then use univariate linear regression to estimate the association of transferring time between pairs of activity states, with BMI. We assign, in turn, one activity state as a baseline and another as a comparison, and calculate the total remaining time per day. The comparison state and remaining time are included in the model and the baseline is not included. For example, we use the following model and multiply $\beta_{1}$ by 10 to estimate the difference in means of BMI for a 10-min per day transfer from the sedentary (baseline) to the moderate (comparison) activity state:

$$BMI\;=\;\beta_{1}\;\times \;M_{d}+\;\beta_{2}\;\times \;r\;+\;\in$$

where $r\;=\;n_{d}-(M_{d}+S_{d})$ and $n_{d}\;$is the number of epochs per day (in this case 1440). We use the number of minutes spent in each activity state in our models rather than the proportion of non-missing time, as we are interested in how the actual amount of time spent in each state associates with BMI. We note that swapping the baseline and comparison activity states results in a reciprocal model with estimate $-\beta_{1}$. We test these associations both before and after adjustment for potential confounders.

#### Modelling activity sequences with activity bigrams

**Figure 2:**
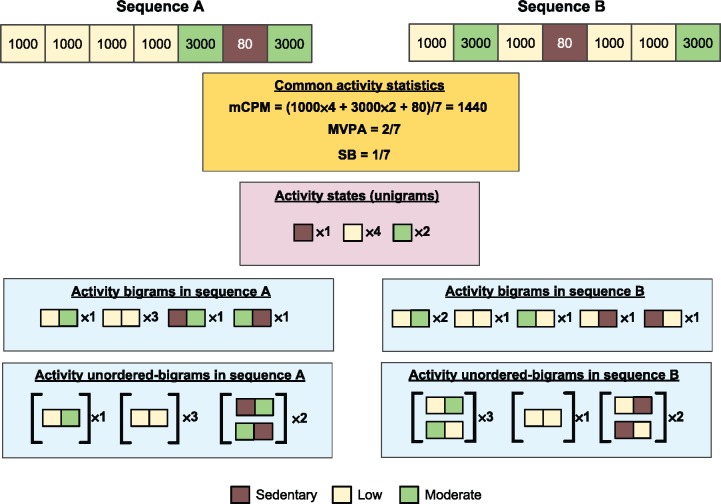
Illustrative examples of common physical activity statistics and our novel activity bigrams. mCPM, average counts per minute; MVPA, proportion of time spent in moderate-vigorous physical activity; SB, sedentary behaviour. Illustration shows two 7-min activity sequences, where each block denotes a 1-min interval with a given activity level. Sequence A and sequence B have the same number of occurrences of each activity state (with the same activity levels) and so have the same values for the common activity statistics and frequency of each activity state, but the different order of activity states means they have different frequencies of bigrams and unordered-bigrams.

We derive a set of variables denoting the number of times a particular bigram occurs in a person’s sequence, on average per day. Given the four activity states—sedentary, low, moderate and vigorous—there are 16 bigrams: SS, SL, SM, SV, LS, LL, LM, LV, MS, ML, MM, MV, VS, VL, VM and VV. For instance, SL denotes the occurrence of the sedentary state at time *t*, followed by the low state at time *t + 1.* In this work, we use a 1-min epoch such that *t* = 1 min. Formally, the frequency of a bigram AB per day is given by:

$$b(A,B)\;=\;\frac{1}{D}\;\underset{i=1:n-1}{\sum }\{\begin{array}{cc} 1,\; & x_{i}=A\wedge x_{i+1}=B\\ 0, & \text{otherwise} \end{array}$$

for number of days *D (*in this work *D = 7)* and sequence x_i_ = {x_1_, x_2_… x_n_} where *x ɛ {S,L,M,V}.*Figure 2 provides two example sequences where the values of the common activity statistics (MVPA, SB and mCPM) are the same, but the frequencies of bigrams differ. For example, the LL bigram occurs three times in sequence A but only once in sequence B. Epoch pairs are overlapping such that a bigram occurring in the time period (t, t + 1) overlaps with the bigrams at (t-1, t) and (t + 1, t + 2). This means that the frequency of each bigram in a sequence does not correspond to a specific amount of time. For instance, the sequences SSSLS and SSLSS both have two occurrences of the SS bigram, but over 3 and 4 min, respectively.

#### Relating activity bigrams to outcomes

**Figure 3:**
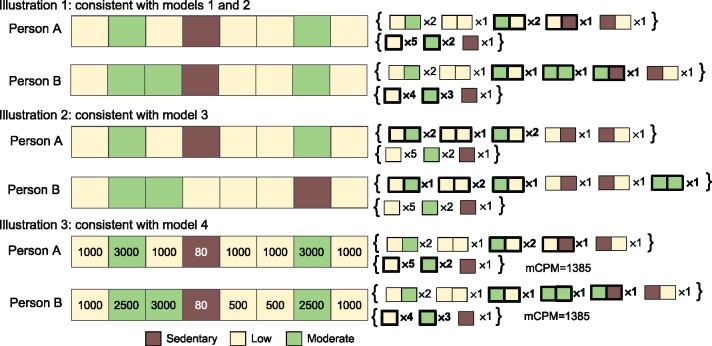
Illustrative examples of real differences in activity sequences consistent with our models, for baseline ML (moderate followed by low) and comparison MM (moderate followed by moderate) activity bigrams. mCPM, average counts per minute. Each illustration shows the activity of two people during an 8-min period, where each block denotes a 1-min interval. Illustration 3 also shows the activity level of each minute. Curly brackets on right-hand side show the frequency of each activity bigram and activity state, where those emboldened have different values for person A and person B. Illustration 1: consistent with models 1 and 2 because swapping ML (the moderate followed by low bigram) with MM (the moderate followed by moderate bigram) increases the occurrence of MM and decreases the occurrence of ML by the same amount. Illustration 2: consistent with model 3 because: 1) sequence changes increase the occurrence of MM and decrease the occurrence of ML by the same amount; and 2) the time spent in sedentary, low, moderate and vigorous does not change. Illustration 3: consistent with model 4 because: 1) swapping ML with MM increases the occurrence of MM and decreases the occurrence of ML by the same amount, and 2) the average counts per minute (mCPM) does not change.

As with activity states, a person can only have a fixed number of occurrences of bigrams in total per day, such that as the frequency of one bigram increases the frequency of at least one other must decrease. Also, because bigrams are overlapping, a change of an epoch pair in a sequence from one bigram to another will often change the number of occurrences of at least one other bigram, and these changes depend on the particular sequence (see examples in Figure 3 and supplementary Section S3, available at *IJE* online). For these reasons, we investigate how BMI changes as the average frequency of bigrams per day increases for one bigram while at the same time decreasing for another bigram, while allowing for collateral changes in other bigrams.

We use univariate linear regression and assign one bigram as a baseline (i.e. not included in the model) and another as a comparison (i.e. included in the model), and adjust for the remaining number of bigrams in a day. For example, we use the following model and multiply $\beta_{1}$ by 10 to estimate the difference in means of BMI for a 10-epoch pair increase of the SL bigram, coupled with a 10-epoch pair decrease of the SS bigram:

$$BMI\;=\;\beta_{1}\;\times \;b(S,L)+\;\beta_{2}\;\times \;r_{b}\;+\;\in$$

where the remaining number of bigrams in a day is given by: $r_{b}\;=\;n_{d}-1-b(S,L)-b(S,S)$. We work with 10 epoch pairs as a non-negligible amount for which a person may reasonably be expected to change their activity. Adjusting for the combined frequency of the remaining bigrams rather than the frequency of each bigram separately, allows for collateral changes in the frequency of these bigrams (while their total frequency remains the same). Whereas we may think of these models as representing a swap from one bigram to another at particular positions in a person’s sequence, in fact any increase in frequency of one bigram that is accompanied by an equal decrease in frequency of another bigram, is consistent with these models (see Supplementary Section S3 for further explanation).

We investigate the impact of potential confounding by characteristics that relate to both the bigram measures and BMI by including these characteristics as covariables in the regression analyses (see Table 1 for confounders). We also consider that the following accelerometer variables might confound associations of bigrams with BMI: mCPM and the number of minutes spent in sedentary, low, moderate or vigorous activity. This is because these will be related to the bigrams and it is well established that they are related to BMI.

**Table 1:** Summary statistics of ALSPAC participants who attended the focus@11 clinic, who are included and not included in our sample

	Attended focus@11 clinic and not in sample		Attended focus@11 clinic and in sample		Difference between participants and non-participants^b^
	Number of participants	Mean (SD) or *N* (%)^a^	Number of participants	Mean (SD) or *N* (%)^a^	Odds ratio (95% CI)
BMI in kg/m^2^ [mean (SD)]	2297	19.39 (3.73)	4810	18.97 (3.30)	0.97 (0.95, 0.98)
**Potential confounding factors**					
Age in years at age 11 clinic [mean (SD)]	2343	11.82 (0.26)	4810	11.77 (0.23)	0.45 (0.36, 0.55)
Parity (%): 0	2343	869 (37.09)	4810	2219 (46.13)	0.66 (0.62, 0.70)
1		619 (26.42)		1688 (35.09)	
2+		855 (36.49)		903 (18.77)	
Sex: % female	2343	1114 (47.55)	4810	2522 (52.43)	1.22 (1.10, 1.34)
Ethnicity: % non-White	1640	82 (5.00)	4810	169 (3.51)	0.69 (0.53, 0.91)
Mother smokes in pregnancy: % yes	1900	455 (23.95)	4810	821 (17.07)	0.65 (0.57, 0.74)
Household social class (%): I	1440	199 (13.82)	4810	776 (16.13)	0.86 (0.81, 0.91)
II		614 (42.64)		2185 (45.43)	
III (non-manual)		358 (24.86)		1215 (25.26)	
III (manual)		180 (12.50)		468 (9.73)	
IV/V		89 (6.18)		166 (3.45)	
Maternal education (%): less than O level	1503	322 (21.42)	4810	877 (18.23)	1.06 (1.00, 1.13)
% O level		530 (35.26)		1788 (37.17)	
% A level		418 (27.81)		1333 (27.71)	
% Degree		233 (15.50)		812 (16.88)	
					

^a^Mean (SD) for continuous and percentage for binary variables.

^b^Odds ratio for participants included in our sample versus participants who attended the age 11 clinic but are not in our sample (reference group), for a one-unit increase in continuous variable (using variable units as described in column 1), or comparison group (indicated in column 1) versus baseline group for binary variables, or a one-category increase for ordinal categorical variables. For example, an odds ratio of 0.97 (0.95, 0.98) for BMI means that on average a participant (who attended the age 11 clinic) is 3% (95% CI: 5%, 2%) less likely to be in our sample for each 1-kg/m^2^ increase in BMI.

These adjustments were made in a series. In all analyses we undertake the following:

- model 1—unadjusted;
- model 2—adjusted for child gender, exact age at age 11 clinic, parity, household social class, maternal education, maternal smoking during pregnancy and child ethnicity;
- model 3 as model 2 and additionally adjusted for the mean number of minutes per day spent in the activity states: sedentary, low, moderate and vigorous;
- model 4—as model 2 and additionally adjusted for mCPM.

Figure 3 provides illustrative examples showing how our models relate to differences in activity sequences (see Supplementary material Section S3 and Supplementary Table 1 for further examples, available at *IJE* online).

#### Relating unordered-bigrams to outcomes

Whereas bigrams denote the ordered occurrence of consecutive activity states, it may be the case that it is the adjacent occurrence of activity states that matters rather than the sequential order. For instance, the frequency of the MV bigram may associate with BMI because M and V are adjacent rather than because V follows M, and where this is true we would expect the associations for MV with BMI to be comparable to the associations for VM with BMI.

We repeat our analyses using an unordered version of bigrams, in order to maximize the power of our analyses, and refer to these as unordered-bigrams (u-bigrams). Given the activity states (sedentary, low, moderate and vigorous), there are 10 u-bigrams: [SS], [SL], [SM], [SV], [LL], [LM], [LV], [MM], [MV] and [VV]. For instance, the [SL] u-bigram corresponds to the bigrams SL and LS, and [VV] corresponds to the bigram VV. Formally the frequency of a u-bigram per day is calculated as:

$$b_{u}\left(A,B\right)\;=\;\frac{1}{D}\;\underset{i=1:n-1}{\sum }\{\begin{array}{cc} 1, & x_{i}=A\wedge x_{i+1}=B\\ 1, & x_{i}=B\wedge x_{i+1}=A\\ 0, & otherwise \end{array}$$

for u-bigram [AB], number of days *D*, and sequence x_i_ = {x_1_, x_2_… x_n_} where *x ɛ {S,L,M,V}.* Clearly, the u-bigrams [SS], [LL], [MM] and [VV] are equivalent to the bigrams SS, LL, MM and VV, respectively. We refer to two bigrams AB and BA, corresponding to the u-bigram [AB], as the reciprocal of each other. Example u-bigrams are given in Figure 2 and example sequence changes consistent with our models are given in Supplementary Table 2, available at *IJE* online.

Summary statistics (Table 1) were generated using Stata SE14. All other analyses are performed in Matlab (R2015). All code is available at [https://github.com/MRCIEU/activityBigrams/]. Git tag v0.3 corresponds to the version presented here.

## Results

Table 1 shows characteristics of participants included in our analysis sample compared with those who were eligible (i.e. attended the age 11 clinic) but were not included in our sample because of missing accelerometer, BMI or confounder data. Participants who were younger, lighter, female, White, with a higher household social class (nearer to class I), higher maternal education and whose mothers did not smoke in pregnancy were more likely to be in our sample (than to have attended the same clinic but not be in our sample), though the magnitudes of these differences were small.

### Associations of conventional summary activity variables (mCPM and time spent in activity states), with BMI

**Figure 4:**
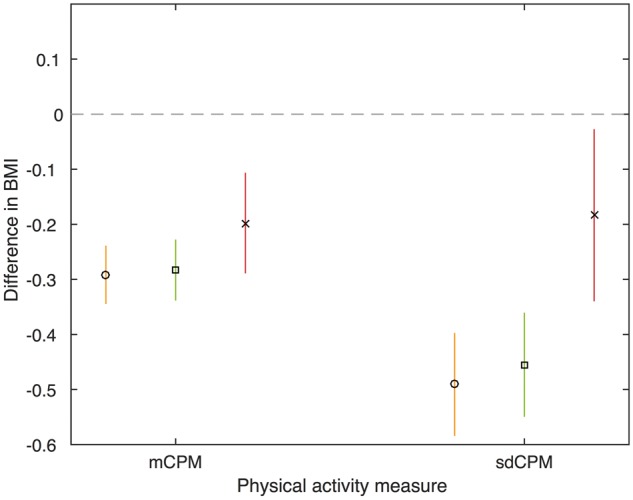
Association of the average counts per minute and standard deviation of counts per minute with BMI. BMI, body mass index; mCPM, average counts per minute; sdCPM, standard deviation of counts per minute. Model 1 (circle): unadjusted. Model 2 (square): adjusted for potential confounders (gender, exact age at age 11 clinic, parity, household social class, maternal education, maternal smoking during pregnancy and child ethnicity). Model 3 (cross): adjusted for potential confounders (gender, exact age at age 11 clinic, parity, household social class, maternal education, maternal smoking during pregnancy and child ethnicity), and mutually adjusted (sdCPM is adjusted for mCPM and vice versa). mCPM estimates are the difference in means of BMI for a 100-counts per minute higher mCPM. sdCPM estimates are the difference in means of BMI for a one-SD higher sdCPM. *N* = 4810.

Before presenting the results of our novel activity bigram variables in the following section, here we present results for the common activity statistics (mCPM and time spent in activity states: sedentary, low, moderate and vigorous) and sdCPM. Table 2 and Figure 4 show the association of mCPM and sdCPM with BMI. The variables mCPM and sdCPM are strongly correlated (Pearson correlation coefficient = 0.80). After adjustment for confounders, a 100 count per minute increase in mCPM is associated with a 0.283-kg/m^2^ lower BMI (95% CI: −0.337, −0.229), and after adjustment for sdCPM this association attenuates slightly towards the null. After adjustment for confounders a 1-SD increase in sdCPM is associated with a 0.455-kg/m^2^ lower BMI (95% CI: −0.549, −0.362). After adjustment for mCPM, this association attenuates considerably towards the null.

**Table 2:** Association of the average counts per minute and variance of counts per minute with BMI

	Difference in means of BMI (kg/m^2^) per unit increase in each activity variable (95% confidence interval) *N* = 4810		
	Model 1 (unadjusted)	Model 2 (non-accelerometer confounder adjusted)	Model 3 (non-accelerometer confounder adjusted and mutually adjusted)
mCPM (per 100 counts)	−0.292 (−0.344, −0.240)	−0.283 (−0.337, −0.229)	−0.198 (−0.288, −0.107)
sdCPM (per 1 SD; 1SD = 347.53 counts/minute)	−0.491 (−0.583, −0.399)	−0.455 (−0.549, −0.362)	−0.184 (−0.339, −0.028)

mCPM, average counts per minute; sdCPM, standard deviation of counts per minute.

Model 1: unadjusted.

Model 2: adjusted for potential confounders (gender, exact age at age 11 clinic, parity, household social class, maternal education, maternal smoking during pregnancy and child ethnicity).

Model 3: adjusted for potential confounders (gender, exact age at age 11 clinic, parity, household social class, maternal education, maternal smoking during pregnancy and child ethnicity), and mutually adjusted (sdCPM is adjusted for mCPM and vice versa).

**Figure 5:**
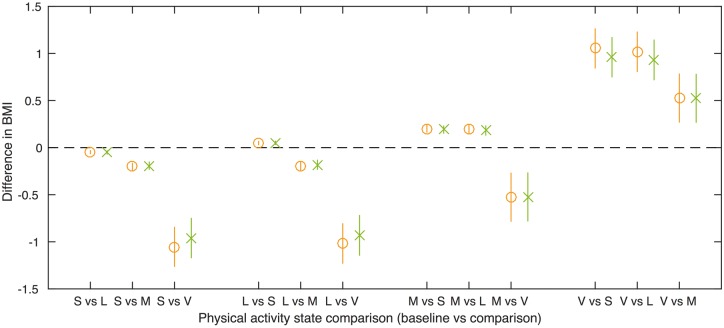
Difference in means of BMI for a 10-occurrences per day transfer from baseline activity state to comparison activity state. Model 1 (circle): unadjusted. Model 2 (cross): adjusted for potential confounders (gender, exact age at age 11 clinic, parity, household social class, maternal education, maternal smoking during pregnancy and child ethnicity). *N* = 4810.

Table 3 and Figure 5 show the unadjusted and confounder-adjusted associations of transferring time between activity states (sedentary, low, moderate and vigorous), with BMI. In general, transferring time to a higher activity state was associated with a lower BMI, and the greater the increase in activity state, the greater the change in BMI. For example, transferring 10 min of time from the sedentary to the vigorous activity state per day was associated with a 0.960-kg/m^2^ lower BMI (95% CI: −1.169, −0.751), after adjustment for non-accelerometer confounders (i.e. model 2).

**Table 3:** Difference in means of BMI for 10-min per day transfer from baseline activity state to comparison activity state, *N* = 4810

Baseline activity state	Model	Comparison activity state					
		Sedentary		Low		Moderate	
		Difference in means of BMI (kg/m^2^)	95% CI	Difference in means of BMI (kg/m^2^)	95% CI	Difference in means of BMI (kg/m^2^)	95% CI
Sedentary	Model 1						
	Model 2						
Low	Model 1	0.048	0.031, 0.065				
	Model 2	0.051	0.034, 0.068				
Moderate	Model 1	0.201	0.166, 0.236	0.196	0.151, 0.241		
	Model 2	0.192	0.154, 0.229	0.181	0.133, 0.230		
Vigorous	Model 1	1.053	0.846, 1.261	1.018	0.809, 1.227	0.526	0.271, 0.782
	Model 2	0.960	0.751, 1.169	0.931	0.721, 1.142	0.524	0.269, 0.778

Model 1: unadjusted.

Model 2: adjusted for potential confounders (gender, exact age at age 11 clinic, parity, household social class, maternal education, maternal smoking during pregnancy and child ethnicity).

Swapping the baseline and comparison activity states gives equivalent associations with BMI (same values but with opposite sign), hence we present only one of each.

### Associations between sequences of physical activity (bigrams and u-bigrams), with BMI

The distributions of the bigrams are described in Table 4 and shown in Supplementary Figure 1, available as Supplementary data at *IJE* online. The frequencies of reciprocal bigrams in an individual’s sequence were highly correlated [Pearson correlation coefficients range from 0.479 (95% CI: 0.457, 0.500) for SV versus VS, to 0.999 (95% CI: 0.999, 0.999) for SL versus LS; see Supplementary Table 3, available as Supplementary data at *IJE* online]. The associations of reciprocal bigrams (such as MV and VM) with BMI were largely consistent (see Supplementary Table 4 and Supplementary Figures 2–17 for bigram results, available as Supplementary data at *IJE* online). For example, a 10-epoch pair higher frequency of MV, coupled with a 10-epoch pair lower frequency of SS, is associated with a 2.308-kg/m^2^ lower BMI (95% CI: −3.553, −1.064), and a 10-epoch pair higher frequency of VM, coupled with a 10-epoch pair lower frequency of SS, is associated with a 1.926-kg/m^2^ lower BMI (95% CI: −3.169, −0.683), after adjustment for confounders and the average frequency of activity states per day (model 3). This suggests that the order of the activity states within a bigram (e.g. MV versus VM) does not affect its association with BMI, and so we present the u-bigram results as the main results.

**Table 4:** Bigram summary statistics

Bigram	Median	Interquartile range
SS	263.43	216.00, 314.57
SL	83.86	73.00, 92.86
SM	2.14	1.57, 2.86
SV	0.00	0.00, 0.14
LS	83.86	73.14, 93.00
LL	219.86	182.86, 256.86
LM	23.00	17.86, 29.14
LV	0.57	0.29, 1.00
MS	2.29	1.57, 3.00
ML	23.00	17.71, 29.14
MM	27.43	17.43, 40.00
MV	1.00	0.43, 2.00
VS	0.00	0.00, 0.14
VL	0.57	0.29, 1.00
VM	1.00	0.43, 2.00
VV	0.43	0.00, 1.43

Bigram distributions are shown in Supplementary Figure 1, available as Supplementary data at *IJE* online.

**Figure 6:**
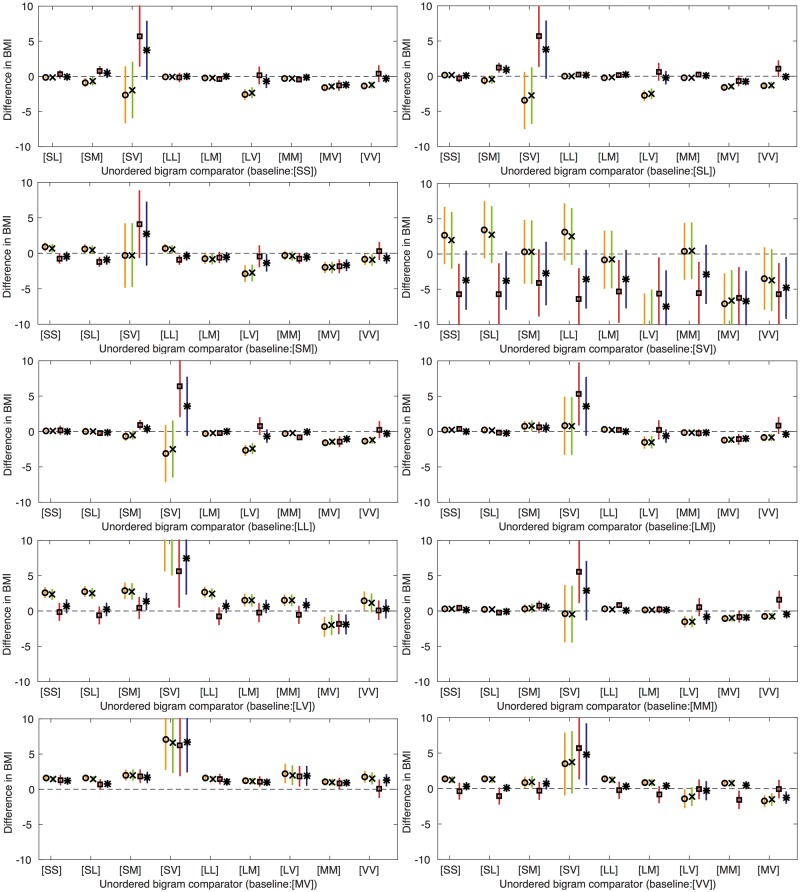
Difference in means of BMI for a 10-epoch pair lower frequency of baseline activity u-bigram, coupled with a 10-epoch pair higher frequency of comparison activity u-bigram. Swapping the comparison and baseline u-bigram gives equivalent estimates of association with BMI (same values but with opposite sign). Model 1 (circle): unadjusted. Model 2 (cross): adjusted for potential confounders (gender, exact age at age 11 clinic, parity, household social class, maternal education, maternal smoking during pregnancy and child ethnicity). Model 3 (square): adjusted for potential confounders (gender, exact age at age 11 clinic, parity, household social class, maternal education, maternal smoking during pregnancy and child ethnicity) and activity states (time spent in sedentary, low, moderate and vigorous activity). Model 4 (star): adjusted for potential confounders (gender, exact age at age 11 clinic, parity, household social class, maternal education, maternal smoking during pregnancy and child ethnicity) and mCPM. *N* = 4810.

Table 5 and Figure 6 show the associations of frequency changes of u-bigrams with BMI (models 1, 2 and 4 are shown in Supplementary Table 5, available as Supplementary data at *IJE* online). An increase in frequency of the [MV] u-bigram, when coupled with a decrease in frequency of all other u-bigrams except [VV], show negative associations with BMI, after adjusting for confounders and also the time spent in each activity state, or mCPM, respectively. For example, a 10-epoch pair higher frequency of [MV] coupled with a 10-epoch pair lower frequency of [SM], is associated with a 1.840-kg/m^2^ lower BMI (95% CI: −2.701, −0.980), after adjustment for confounders and the time spent in each activity state. We also found associations for an increase in frequency of the [MM] u-bigram, when coupled with a decrease in frequency of the [SS] and [LL] u-bigrams, which remained after adjusting for both the time spent in each activity state, and mCPM, respectively.

**Table 5:** Difference in means of BMI for a 10-epoch pair lower frequency of baseline u-bigram, coupled with a 10-epoch pair higher frequency of comparison u-bigram, after adjustment for confounding factors and amount of time spent in each activity state (model 3)

Baseline u-bigram	Comparison u-bigram								
	[SS]	[SL]	[SM]	[SV]	[LL]	[LM]	[LV]	[MM]	[MV]
			Difference in means of BMI (95% CI) (*N* = 4810)				
[SL]	−0.267								
	(−0.766, 0.232)								
[SM]	−0.774	−1.204							
	(−1.348, −0.199)	(−1.789, −0.620)							
[SV]	−5.739	−5.680	−4.107						
	(−9.986, −1.492)	(−9.927, −1.434)	(−8.768, 0.553)						
[LL]	0.168	−0.255	0.948	6.389					
	(−0.380, 0.717)	(−0.418, −0.092)	(0.372, 1.523)	(2.141, 10.638)					
[LM]	0.411	−0.188	0.580	5.319	0.242				
	(0.206, 0.616)	(−0.320, −0.055)	(−0.112, 1.272)	(0.971, 9.666)	(0.075, 0.409)				
[LV]	−0.130	−0.625	0.423	5.642	−0.744	−0.234			
	(−1.289, 1.030)	(−1.775, 0.524)	(−1.021, 1.867)	(0.586, 10.698)	(−1.894, 0.405)	(−1.489, 1.022)			
[MM]	0.479	−0.203	0.753	5.538	0.852	0.208	0.546		
	(0.232, 0.727)	(−0.472, 0.065)	(0.167, 1.340)	(1.232, 9.844)	(0.564, 1.141)	(−0.218, 0.634)	(−0.618, 1.709)		
[MV]	1.307	0.687	1.840	6.234	1.442	1.101	1.836	0.839	
	(0.664, 1.950)	(0.040, 1.334)	(0.980, 2.701)	(1.966, 10.502)	(0.793, 2.090)	(0.457, 1.744)	(0.491, 3.181)	[0.166, 1.513]	
[VV]	−0.376	−1.058	−0.322	5.671	−0.264	−0.872	−0.109	−1.588	−0.075
	(−1.467, 0.715)	(−2.136, 0.021)	(−1.487, 0.842)	(1.396, 9.945)	(−1.362, 0.834)	(−1.959, 0.215)	(−1.382, 1.165)	[−2.779, −0.397]	[−1.277, 1.126]

Swapping the comparison and baseline u-bigram gives equivalent estimates of association with BMI (same values but with opposite sign), hence we present only one of each.

S, sedentary; L, low; M, moderate; V, vigorous; u-bigram, unordered bigram.

Model 3 is adjusted for potential confounders (gender, exact age at age 11 clinic, parity, household social class, maternal education, maternal smoking during pregnancy and child ethnicity) and activity states (time spent in sedentary, low, moderate and vigorous activity).

Full results (models 1–4) are given in Supplementary Table 5, available as Supplementary data at *IJE* online.

Although we present associations for a 10-frequency change, this may not represent feasible changes in activity for all u-bigrams, as their standard deviations vary widely (from 1.66 to 526.64 epoch pairs for the [SV] and [SS] u-bigrams, respectively; see Supplementary Table 6, available as Supplementary data at *IJE* online). Hence, whereas the large estimates for [SV] in our main analysis appear unfeasible, this is because occurrences of [SV] are infrequent. Supplementary Table 7 and Supplementary Figure 18, available as Supplementary data at *IJE* online, present associations for a 1-SD change of baseline bigram frequency, reflecting a realistic change based on variation in frequency of bigrams across our sample.

Our analysis used all 7 days of accelerometer data, including those with less than 8 h of wear time. We performed a sensitivity analysis to check the impact of this on our results, by including only days with at least 8 h wear time in analyses, and this gave results consistent with those reported here (see Supplementary Table 8 and Supplementary Figure 19, available as Supplementary data at *IJE* online).

## Discussion

In this work we have shown how activity bigrams can be used to investigate changes in activity from one moment to the next, and how these can then be used to assess the associations of finely graded patterns of change in activity across a day with disease- or health-related traits such as BMI. Reciprocal bigrams (with the same sets of activity states, e.g. MV and VM) had comparable associations with BMI. This may be because these bigrams correlate highly with each other; for example, people with more occurrences of the MV bigram on average have more VM bigrams.

Our tests of association of the u-bigrams with BMI identified several sequential activity patterns that were associated with BMI. In particular, a higher frequency of the MV u-bigram, coupled with a lower frequency of all other u-bigrams except [VV] was associated with a lower BMI, even after adjusting for mCPM and the amount of time spent in each activity state (sedentary, low, moderate and vigorous), respectively. This indicates that, given two groups of people who spent the same amount of time in each activity state per day and the same number of adjacent minutes in the vigorous state, those who have more adjacent minutes of moderate and vigorous, have a lower BMI. Hence, whereas current physical activity recommendations say adults should do at least 150 min of moderate- or 75 min of vigorous-intensity physical activity a week,^19^^,^^26^^,^^27^ it may also be important to consider how activity levels change from one moment to the next. For instance, it might be that frequent occurrences of the acute increase in heart rate and changes to metabolism that occur with consecutive minutes in moderate and vigorous activity are important for lowering BMI.

Thus, if further research replicates our findings and demonstrates similar associations with other health-related outcomes, and evidence suggests these associations are causal, then public health advice in relation to physical activity might need to change. To date, analysis and hence advice on physical activity have focused on average levels of activity. Exploring associations between sequential patterns of activity with other traits and disease will enable more comprehensive advice about the types and patterns of change in activity that may be beneficial or detrimental to health. For example, if a causal effect of adjacent minutes in moderate and vigorous activity on BMI was established and extended to obesity-related disease outcomes such as diabetes and cardiovascular disease, it may be appropriate to advise performing moderate and vigorous activity sequentially, rather than separately throughout the day.

We also investigated whether the standard deviation of activity levels (sdCPM) was associated with BMI, and found evidence of an association conditional on mCPM.

### Study limitations

We use the average frequency of bigrams over 7 days as independent variables in linear regression models, without considering the uncertainty in these estimates. Therefore the confidence intervals of the associations with BMI may be underestimated. Estimates based on a larger number of days may improve the accuracy of the bigram variables and hence the accuracy of associations based on these estimates. We identified differences in characteristics between ALSPAC participants included in our sample, and those who attended the age 11 clinic but were not included in our sample. This may bias associations if these data are not missing at random. However, the magnitudes of the differences were small and hence major bias unlikely. This paper is primarily concerned with demonstrating a novel (activity bigram) method. In future more applied papers, we would want to undertake sensitivity analyses to explore the likelihood that our assumptions about missing data are correct.

We cannot infer that the associations with BMI we have presented in this paper are causal. Associations may be because the bigram (or u-bigram) has a causal effect on BMI. Alternatively, it may be the case that people with higher BMI are less likely to partake in activities that involve this type of activity pattern (i.e. more obese people may be less likely to change frequently from moderate to vigorous activity). Finally, although we adjusted for common confounding factors, it is possible that associations may be due to residual confounding.

We note that our analysis used a 1-min epoch such that the bigrams are a sequence of two 1-min intervals. The association of a bigram with another trait is likely to change as the epoch size changes. For example, a 1-min epoch of moderate activity may be composed of one 30-s interval at low and one at vigorous activity, rather than continuous activity at the moderate level. The accelerometers used in ALSPAC measured data in 1-min intervals, but accelerometers are increasingly being used to collect raw data at a much higher resolution. Our methods are applicable to such data and could be used to determine whether associations with health/disease-related traits differ with different epoch sizes. Also, whereas in this work we have used activity bigrams, it is possible to extend this approach to other *n*-grams. However, as *n* increases, the number of occurrences of each *n*-gram in the population decreases and hence so does the study power.

To conclude, we have shown how a method initially developed for text data mining can be used with accelerometer data to explore whether variation in physical activity intensity from one moment to the next, over and above mean levels of time spent at a given intensity, relates to health outcomes. We have shown that for BMI and activity bigrams calculated using a 1-min epoch, this does appear to be the case. We recommend that other studies explore whether our findings with BMI replicate, and that the association of activity bigrams with other traits are assessed.

## Supplementary Data

Supplementary data are available at *IJE* online.

## Funding

This work was supported by the University of Bristol and UK Medical Research Council [grant numbers MC_UU_12013/5, MC_UU_12013/8 and MC_UU_12013/9]. D.A.L. is a National Institute of Health Research Senior Investigator [grant number NF-SI-0166‐10196]. The UK Medical Research Council and the Wellcome Trust (grant ref: 102215/2/13/2) and the University of Bristol provide core support for ALSPAC.

## Supplementary Material

Supplementary Data

## References

[1] American Diabetes Association. Physical activity/exercise and diabetes. Diabetes Care 2004;27 **(****Suppl 1****):** s58–62. 14693927 10.2337/diacare.27.2007.s58

[2] Berlin J, Colditz G A meta-analysis of physical activity in the prevention of coronary heart disease. Am J Epidemiol 1990;132**:** 612–28. 2144946 10.1093/oxfordjournals.aje.a115704

[3] Sallis J, Saelens B Assessment of physical activity by self-report: status, limitations, and future directions. Res Q Exerc Sport 2000;71 **(****Suppl 2****):** 1–14. 10.1080/02701367.2000.11082780 25680007

[4] UK Biobank. Physical Activity Monitor (Accelerometer), *version 1.0*. 2016 http://biobank.ctsu.ox.ac.uk/showcase/refer.cgi?id=131600 (17 May 2017, date last accessed).

[5] Riddoch C, Mattocks C, Deere K Objective measurement of levels and patterns of physical activity. Arch Dis Child 2007;92**:** 963–69. 17855437 10.1136/adc.2006.112136 PMC2083612

[6] Healy GN, Matthews CE, Dunstan DW Sedentary time and cardio-metabolic biomarkers in US adults: NHANES 2003–06. Eur Heart J 2011;32**:** 590–97. 21224291 10.1093/eurheartj/ehq451 PMC3634159

[7] Leary SD, Ness AR, Smith GD Physical activity and blood pressure in childhood. Hypertension 2008;51**:** 92–98. 18071055 10.1161/HYPERTENSIONAHA.107.099051

[8] Ekelund U, Luan J, Sherar LB Moderate to vigorous physical activity and sedentary time and cardiometabolic risk factors in children and adolescents. JAMA 2012;307**:** 704–12. 22337681 10.1001/jama.2012.156 PMC3793121

[9] Luke A, Dugas LR, Durazo-Arvizu RA Assessing physical activity and its relationship to cardiovascular risk factors: NHANES 2003–2006. BMC Public Health 2011;11**:** 387. 21612597 10.1186/1471-2458-11-387 PMC3123595

[10] Wiles NJ, Haase AM, Lawlor DA, Ness A, Lewis G Physical activity and depression in adolescents: cross-sectional findings from the ALSPAC cohort. Soc Psychiatry Psychiatr Epidemiol 2011;47**:** 1023–33. 21826444 10.1007/s00127-011-0422-4 PMC3382270

[11] Cliff DP, Hesketh KD, Vella SA Objectively measured sedentary behaviour and health and development in children and adolescents: systematic review and meta‐analysis. Obes Rev 2016;17**:** 330–44. 26914664 10.1111/obr.12371

[12] Stamatakis E, Coombs N, Tilling K Sedentary time in late childhood and cardiometabolic risk in adolescence. Pediatrics 2015;135**:** e1432–41. 25986017 10.1542/peds.2014-3750 PMC4444802

[13] Goldsmith J, Liu X, Rundle A, Jacobson J New insights into activity patterns in children, found using functional data analyses. Med Sci Sport Exerc 2016;48**:** 1723–29. 10.1249/MSS.0000000000000968 PMC4987214 27183122

[14] Evenson KR, Wen F, Hales D, Herring AH National youth sedentary behavior and physical activity daily patterns using latent class analysis applied to accelerometry. Int J Behav Nutr Phys Act 2016;13**:** 55. 27142304 10.1186/s12966-016-0382-x PMC4855777

[15] Augustin NH, Mattocks C, Faraway JJ, Greven S, Ness AR Modelling a response as a function of high frequency count data: the association between physical activity and fat mass. Stat Methods Med Res 2015, Jul 17. pii: 0962280215595832. [Epub ahead of print.] 10.1177/0962280215595832 26187735

[16] Rothwell PM, Howard SC, Dolan E Prognostic significance of visit-to-visit variability, maximum systolic blood pressure, and episodic hypertension. Lancet 2010;375**:** 895–905. 20226988 10.1016/S0140-6736(10)60308-X

[17] Rothwell PM, Howard SC, Dolan E Effects of β blockers and calcium-channel blockers on within-individual variability in blood pressure and risk of stroke. Lancet Neurol 2010;9**:** 469–80. 20227347 10.1016/S1474-4422(10)70066-1

[18] Rothwell P Limitations of the usual blood-pressure hypothesis and importance of variability, instability, and episodic hypertension. Lancet 2010;375**:** 938–48. 20226991 10.1016/S0140-6736(10)60309-1

[19] Department of Health. UK Physical Activity Guidelines. London: DoH, 2011.

[20] Physical Activity Guidelines Advisory Committee. Physical Activity Guidelines for Americans. Washington, DC: US Department of Health and Human Services, 2008.

[21] Hood MS, Little JP, Tarnopolsky MA Low-volume interval training improves muscle oxidative capacity in sedentary adults. Med Sci Sports Exerc 2011;43**:** 1849–56. 21448086 10.1249/MSS.0b013e3182199834

[22] Little JP, Gillen JB, Percival ME Low-volume high-intensity interval training reduces hyperglycemia and increases muscle mitochondrial capacity in patients with type 2 diabetes. J Appl Physiol 2011;111**:** 1554–60. 21868679 10.1152/japplphysiol.00921.2011

[23] Wisløff U, Støylen A, Loennechen JP Superior cardiovascular effect of aerobic interval training versus moderate continuous training in heart failure patients: a randomized study. Circulation 2007;115**:** 3086–94. 17548726 10.1161/CIRCULATIONAHA.106.675041

[24] Hotho A, Nürnberger A, Paaß G A brief survey of text mining*. *LDV Forum – GLDV J Comput Linguist Lang Technol 2005;20**:** 19–62.

[25] Boyd A, Golding J, Macleod J Cohort Profile: The ‘children of the 90s’—the index offspring of the Avon Longitudinal Study of Parents and Children. Int J Epidemiol 2012;42**:** 111–27. 22507743 10.1093/ije/dys064 PMC3600618

[26] Tucker JM, Welk GJ, Beyler NK Physical activity in U.S.: adults compliance with the Physical Activity Guidelines for Americans. Am J Prev M 2011;40**:** 454–61. 21406280 10.1016/j.amepre.2010.12.016

[27] Brown W, Bauman A, Bull F, Burton N Development of Evidence-based Physical Activity Recommendations for Adults (18–64 Years). Canberra: Australian Government Department of Health, 2013.

