# Supplementary material for: Physical activity phenotyping with activity bigrams, and their association with BMI
Source: Int J Epidemiol. 2017 Jun 29;46(6):1857–70. doi: 10.1093/ije/dyx093 (PMC5837541; doi:10.1093/ije/dyx093)
Supplement: Supplementary Data [file ije-2016-10-1225-file008_dyx093.pdf]

Physical activity phenotyping with activity bigrams, and their association with BMI

Louise AC Millard [*1,2,3], Kate Tilling [1,2], Debbie A Lawlor [1,2], Peter A Flach [1,3], Tom R Gaunt [1,2]

[*] Corresponding author: louise.millard@bristol.ac.uk

[1] MRC Integrative Epidemiology Unit (IEU), University of Bristol, Bristol, United Kingdom

[2] School of Social and Community Medicine, University of Bristol, Bristol, United Kingdom

[3] Intelligent Systems Laboratory, University of Bristol, Bristol, United Kingdom

SUPPLEMENTARY MATERIAL

Supplementary section S1: Bags of n-grams

An n-gram is a contiguous sequence of length n. The 1-gram and 2-gram are referred to as unigrams and bigrams, respectively.

A bag on n-grams is the set of n-grams and associated frequency, from a given sequence. For example, the sequence ABAB has 2 A and 2 B characters and so can be represented by the bag of unigrams {A*2, B*2}. This sequence can also be represented by bags of bigrams {AB*2, BA}, 3-grams {ABA, BAB} and 4-grams {ABAB}.

Bags of unigrams represent any sequence that is a permutation of the elements in the bag. For example, the bag of unigrams {A*2, B*2} can represent the sequences AABB, ABAB, ABBA, and so forth. Where n>1, n-grams are overlapping such that, for instance, the bigram at position [i, i+1] overlaps with the bigrams at positions [i-1, i] and [i+1, i+2]. This restricts the possible sequences that each bag of n-grams represents. For example, the bag of bigrams {AB*2, BA} represents the sequence ABAB only. The bag of bigrams {AA*2, AB, BB, BA} represents the sequences AAABBA, ABBAAA and BBAAAB.

Supplementary section S2: Potential confounders

Parity was recorded via questionnaire at 18 weeks gestation. We derived a measure of maternal smoking in pregnancy using questionnaire measures recorded at 18 and 32 weeks gestation. At 18 weeks gestation the mother was asked if they 1) smoked tobacco in the first 3 months of pregnancy or 2) smoked tobacco in the last 2 weeks. At 32 weeks gestation she was asked how many cigarettes she was currently smoking per day.

At 32 weeks gestation each mother was asked to record their highest education level, as either none, CSE (national school exams at age 16), or vocational; O level (national school exams at age 16, higher than CSE); A level (national school exams at age 18); or university degree. We combined the categories ‘CSE’ and ‘vocational’ into a single category due to the small number of participants in these groups. The mother also recorded her and her partner’s occupation and these were used to derive their social class groups (I, II, III manual, III non-manual, IV, V) using the 1991 Office of Population, Censuses and Surveys classification. The lowest of the mothers or her partner’s social class was used as a measure of the household social class. The mother was also asked about their and their partner’s ethnicity and this was used to derive the child’s ethnicity.

Supplementary section S3: Relating our linear models to real changes in activity sequences

Figure 3 in the main paper shows three example sequence changes that are consistent with particular models in our analyses. In this section we provide further explanation and examples of sequence changes that are consistent with our models.

As discussed in the main paper, bigrams are overlapping such that a change of one epoch pair in a sequence from one bigram to another will often change the number of occurrences of at least one other bigram. In a minority of cases only, changing an

epoch pair from one bigram to another results in the same bag of bigrams. For example, given the sequence SSLS, changing the SL to LS produces the sequence SLSS. Both before and after this change, the sequence has bag of bigrams {SS, SL, LS}. It is more common that a change of an epoch pair in a sequence from one bigram to another will change the number of occurrences of at least one other bigram, and these changes depend on the particular sequence.

The following two examples both change one occurrence of SS to SM:

Example 1: Sequence SSSLML changes to SSMLML
Example 2: Sequence LMVSSV changes to LMVSMV

In example 1 this change simultaneously adds a second ML bigram and removes the SL bigram, whereas in example 2 this change simultaneously adds a second MV bigram and removes the SV bigram. The actual frequencies of activity states and bigrams are shown in Supplementary table 1.

The sequence changes of examples 1 and 2 are consistent with models 1 and 2 of our analyses. This is because models 1 and 2 corresponds to real sequence changes where there is an increase in frequency of the comparison bigram (SM) and an equivalent decrease of the baseline bigram (SS), such that the total remaining number of bigrams in the sequence (1339 – SS - SM) remains constant.

While we may think of these models as representing a swap from one bigram to another at particular positions in a person's sequence, in fact any increase in frequency of one bigram that is accompanied by an *equal* decrease in frequency of another bigram, is consistent with these models. For instance, swapping SS for SM in example 1 collaterally increases the frequency of ML by 1. Hence this example is also consistent with a model with baseline SS and comparison ML, because the frequency of these bigrams decreases and increases, respectively, by the same amount.

Models 3 and models 4 adjust for the common activity statistics; the frequency of activity states and the average activity, respectively. To be consistent with these models a change in a sequence has to additionally satisfy other properties.

Model 3 represents a change in a sequence where the frequency of one bigram increases, accompanied by an equal decrease in frequency of another bigram, while the time spent in each activity state (sedentary, low, moderate and vigorous) remains constant. Example 1 and 2 described above are examples where the time spent in activity states are not kept constant as, for instance, in example 1 the time spent in the sedentary state decreases by one minute and the time spent in the moderate state increases by one minute. Hence, these examples are not consistent with model 3. The following examples have the same initial sequences as examples 1 and 2 and show a change of one SS to SM, but are consistent with model 3:

Example 3: Sequence SSSLML changes to SSMLSL
Example 4: Sequence LMVSSV changes to LSVSMV

In order to keep the frequency of each activity state unchanged a moderate state has additionally been changed to sedentary (see Supplementary table 1 for more details of these examples).

Model 4 represents a change in a sequence where the frequency of one bigram increases, accompanied by an equal decrease in frequency of another bigram, while the average activity levels do not change. Hence, a change in the frequency of each activity state is consistent, as long as the average activity levels remain the same (as shown in illustration 3 of Figure 2 in the main paper).

While in this section we have focussed on changes in the frequency of bigrams consistent with our models, the same also applies to unordered bigrams (u-bigrams). Supplementary table 2 shows two example u-bigram changes consistent with models 1 and 2, and two examples consistent with model 3.

Supplementary table 1: Examples of changing one SS bigram occurrence to SM with associated changes in bigrams

**Examples of changing a bigram, with associated changes in the frequency other bigrams and activity states (consistent with models 1 and 2)**						
	**Example 1**			**Example 2**		
	**Original sequence**	**Sequence after change**		**Original sequence**	**Sequence after change**	
	**SSSLML**	**SSMLML**		**LMVSSV**	**LMVSMV**	
Bigram	Frequency of each bigram		Change in frequency	Frequency of each bigram		Change in frequency
SS	**2**	**1**	**-1**	**1**	**0**	**-1**
SL	**1**	**0**	**-1**	0	0	0
SM	**0**	**1**	**+1**	**0**	**1**	**+1**
SV	0	0	0	**1**	**0**	**-1**
LS	0	0	0	0	0	0
LL	0	0	0	0	0	0
LM	1	1	0	1	1	0
LV	0	0	0	0	0	0
MS	0	0	0	0	0	0
ML	**1**	**2**	**+1**	0	0	0
MM	0	0	0	0	0	0
MV	0	0	0	**1**	**2**	**+1**
VS	0	0	0	1	1	0
VL	0	0	0	0	0	0
VM	0	0	0	0	0	0
VV	0	0	0	0	0	0
Activity state	Frequency of each activity state		Change in frequency	Frequency of each activity state		Change in frequency
S	3	2	-1	2	1	-1
L	2	2	0	1	1	0
M	1	2	1	1	2	+1
V	0	0	0	2	2	0
**Examples of changing a bigram, with associated changes in the frequency other bigrams, when keeping the frequency of activity**						

states constant (consistent with model 3)						
	**Example 3**			**Example 4**		
	**Original sequence**	**Sequence after change**		**Original sequence**	**Sequence after change**	
	**S<u>SS</u>L<u>M</u>L**	**S<u>SM</u>L<u>S</u>L**		**LMV<u>SS</u>V**	**L<u>S</u>V<u>SM</u>V**	
Bigram	Frequency of each bigram		Change in frequency	Frequency of each bigram		Change in frequency
SS	**2**	**1**	**-1**	**1**	**0**	**-1**
SL	1	1	0	0	0	0
SM	**0**	**1**	**+1**	**0**	**1**	**+1**
SV	0	0	0	1	1	0
LS	**0**	**1**	**+1**	**0**	**1**	**+1**
LL	0	0	0	0	0	0
LM	**1**	**0**	**-1**	**1**	**0**	**-1**
LV	0	0	0	0	0	0
MS	0	0	0	0	0	0
ML	1	1	0	0	0	0
MM	0	0	0	0	0	0
MV	0	0	0	1	1	0
VS	0	0	0	1	1	0
VL	0	0	0	0	0	0
VM	0	0	0	0	0	0
VV	0	0	0	0	0	0
Activity state	Frequency of each activity state		Change in frequency	Frequency of each activity state		Change in frequency
S	3	3	0	2	2	0
L	2	2	0	1	1	0
M	1	1	0	1	1	0
V	0	0	0	2	2	0

S: sedentary; L: low; M: moderate; V: vigorous.

Underlined activity states in sequences shows position at which states change.

Examples 1 and 2 are consistent with models 1 and 2 because the frequency of SM increases by the same amount that SS decreases.
Examples 3 and 4 are consistent with model 3 (adjusted for activity states; sedentary, low, moderate and vigorous) because: 1) the frequency of SM increases by the same amount that SS decreases, and 2) the time spent in sedentary, low, moderate and vigorous activity does not change.

Supplementary table 2: Examples of changing one SS occurrence to SM with associated changes in u-bigrams

**Examples of changing a u-bigram, with associated changes in the frequency other u-bigrams and activity states (consistent with models 1 and 2)**						
	**Example 1**			**Example 2**		
	**Original sequence**	**Sequence after change**		**Original sequence**	**Sequence after change**	
	**SSSLML**	**SSMLML**		**LMVSSV**	**LMVSMV**	
U-bigram	Frequency of each u-bigram		Change in frequency	Frequency of each u-bigram		Change in frequency
[SS]	**2**	**1**	**-1**	**1**	**0**	**-1**
[SL]	**1**	**0**	**-1**	0	0	0
[SM]	**0**	**1**	**+1**	**0**	**1**	**+1**
[SV]	0	0	0	**2**	**1**	**-1**
[LL]	0	0	0	0	0	0
[LM]	**2**	**3**	**+1**	1	1	0
[LV]	0	0	0	0	0	0
[MM]	0	0	0	0	0	0
[MV]	0	0	0	**1**	**2**	**+1**
[VV]	0	0	0	0	0	0
Activity state	Frequency of each activity state		Change in frequency	Frequency of each activity state		Change in frequency
S	3	2	-1	2	1	-1
L	2	2	0	1	1	0
M	1	2	+1	1	2	+1
V	0	0	0	2	2	0
**Examples of changing a u-bigram, with associated changes in the frequency other u-bigrams, when keeping the frequency of activity states constant (consistent with model 3)**						
	**Example 3**			**Example 4**		
	**Original sequence**	**Sequence after change**		**Original sequence**	**Sequence after change**	
	**SSSLML**	**SSMLSL**		**LMVSSV**	**LSVSMV**	
U-Bigram	Frequency of each u-bigram		Change in frequency	Frequency of each u-bigram		Change in frequency

[SS]	**2**	**1**	**-1**	**1**	**0**	**-1**
[SL]	**1**	**2**	**+1**	**0**	**1**	**+1**
[SM]	**0**	**1**	**+1**	**0**	**1**	**+1**
[SV]	0	0	0	2	2	0
[LL]	0	0	0	0	0	0
[LM]	**2**	**1**	**-1**	**1**	**0**	**-1**
[LV]	0	0	0	0	0	0
[MM]	0	0	0	0	0	0
[MV]	0	0	0	1	1	0
[VV]	0	0	0	0	0	0
Activity state	Frequency of each activity state		Change in frequency	Frequency of each activity state		Change in frequency
S	3	3	0	2	2	0
L	2	2	0	1	1	0
M	1	1	0	1	1	0
V	0	0	0	2	2	0

S: sedentary; L: low; M: moderate; V: vigorous; u-bigram: unordered bigram.

Underlined activity states in sequences shows position at which states change.

Examples 1 and 2 are consistent with models 1 and 2 because the frequency of [SM] u-bigram increases by the same amount that [SS] u-bigram decreases.

Examples 3 and 4 are consistent with model 3 (adjusted for activity states; sedentary, low, moderate and vigorous) because: 1) the frequency of [SM] u-bigram increases by the same amount that [SS] u-bigram decreases, and 2) the time spent in sedentary, low, moderate and vigorous activity does not change.

Supplementary table 3: Correlation between reciprocal bigrams of each u-bigram

U-bigram	Constituent bigrams	Correlation	
		Pearson correlation coefficient	95% CI
[SL]	SL, LS	0.999	0.999, 0.999
[SM]	SM, MS	0.710	0.696, 0.724
[SV]	SV, VS	0.479	0.457, 0.500
[LM]	LM, ML	0.996	0.995, 0.996
[LV]	LV, VL	0.886	0.880, 0.892
[MV]	MV, VM	0.982	0.981, 0.983

Correlation calculated with `corrcoef` Matlab function.

S: sedentary; L: low; M: moderate; V: vigorous; CI: confidence interval; u-bigram: unordered bigram.

Supplementary table 4: Difference in means of BMI for a 10 epoch pair lower frequency of baseline bigram, coupled with a 10 epoch pair higher frequency of comparison bigram

Baseline bigram	Comparison bigram	Model 1	Model 2	Model 3	Model 4
		Mean difference in BMI [95% CI] (N=4810)			
SS	SL	-0.223 [-0.293, -0.154]	-0.229 [-0.297, -0.160]	-0.425 [-0.539, -0.311]	-0.200 [-0.269, -0.131]
	SM	-1.129 [-2.070, -0.188]	-0.762 [-1.696, 0.173]	2.113 [1.074, 3.152]	1.249 [0.220, 2.277]
	SV	-5.448 [-12.067, 1.172]	-4.764 [-11.325, 1.796]	6.320 [-0.680, 13.319]	3.409 [-3.362, 10.180]
	LS	-0.220 [-0.289, -0.150]	-0.224 [-0.293, -0.155]	-0.418 [-0.532, -0.305]	-0.196 [-0.264, -0.127]
	LL	-0.041 [-0.060, -0.023]	-0.045 [-0.064, -0.027]	-0.168 [-0.717, 0.380]	0.000 [-0.021, 0.021]
	LM	-0.472 [-0.579, -0.365]	-0.414 [-0.525, -0.304]	-0.460 [-0.786, -0.134]	-0.051 [-0.223, 0.120]
	LV	-4.558 [-5.837, -3.280]	-4.009 [-5.287, -2.731]	1.257 [-0.831, 3.344]	-0.734 [-2.373, 0.904]
	MS	-1.863 [-2.801, -0.924]	-1.646 [-2.576, -0.715]	0.974 [-0.065, 2.012]	0.206 [-0.824, 1.236]
	ML	-0.458 [-0.565, -0.352]	-0.399 [-0.510, -0.288]	-0.361 [-0.686, -0.036]	-0.018 [-0.189, 0.153]
	MM	-0.289 [-0.341, -0.236]	-0.272 [-0.329, -0.215]	-0.479 [-0.727, -0.232]	-0.126 [-0.229, -0.023]
	MV	-3.135 [-3.661, -2.609]	-2.872 [-3.414, -2.330]	-2.308 [-3.553, -1.064]	-2.420 [-3.269, -1.570]
	VS	-2.122 [-9.075, 4.831]	-0.754 [-7.653, 6.146]	10.182 [2.969, 17.396]	7.471 [0.393, 14.549]
	VL	-5.248 [-6.554, -3.941]	-4.700 [-6.004, -3.396]	-0.305 [-2.445, 1.835]	-1.724 [-3.405, -0.044]
	VM	-3.031 [-3.552, -2.510]	-2.772 [-3.310, -2.234]	-1.926 [-3.169, -0.683]	-2.219 [-3.060, -1.377]
	VV	-1.335 [-1.707, -0.963]	-1.242 [-1.611, -0.872]	0.376 [-0.715, 1.467]	-0.324 [-0.799, 0.151]
SL	SS	0.223 [0.154, 0.293]	0.229 [0.160, 0.297]	0.425 [0.311, 0.539]	0.200 [0.131, 0.269]
	SM	-0.694 [-1.655, 0.266]	-0.338 [-1.293, 0.617]	2.527 [1.475, 3.579]	2.089 [1.066, 3.113]
	SV	-6.655 [-13.281, -0.029]	-5.967 [-12.538, 0.605]	6.106 [-0.891, 13.103]	3.623 [-3.079, 10.325]
	LS	2.371 [0.018, 4.725]	2.872 [0.539, 5.206]	2.723 [0.430, 5.017]	2.743 [0.439, 5.047]

	LL	0.059 [-0.020, 0.139]	0.050 [-0.030, 0.131]	0.440 [0.320, 0.560]	0.324 [0.234, 0.415]
	LM	-0.406 [-0.530, -0.282]	-0.365 [-0.495, -0.235]	0.343 [0.080, 0.607]	0.389 [0.172, 0.606]
	LV	-4.880 [-6.144, -3.615]	-4.376 [-5.642, -3.110]	1.895 [-0.184, 3.974]	0.093 [-1.504, 1.689]
	MS	-1.446 [-2.406, -0.487]	-1.246 [-2.197, -0.295]	1.564 [0.506, 2.622]	1.081 [0.055, 2.107]
	ML	-0.393 [-0.516, -0.269]	-0.349 [-0.479, -0.220]	0.412 [0.149, 0.674]	0.430 [0.214, 0.647]
	MM	-0.184 [-0.261, -0.107]	-0.177 [-0.259, -0.096]	0.426 [0.126, 0.727]	0.133 [0.011, 0.256]
	MV	-3.211 [-3.731, -2.692]	-2.968 [-3.505, -2.431]	-1.467 [-2.727, -0.207]	-1.605 [-2.398, -0.813]
	VS	-3.378 [-10.340, 3.585]	-1.951 [-8.865, 4.963]	10.108 [2.897, 17.318]	7.531 [0.519, 14.542]
	VL	-5.577 [-6.870, -4.285]	-5.083 [-6.375, -3.792]	0.286 [-1.846, 2.418]	-0.873 [-2.507, 0.761]
	VM	-3.104 [-3.618, -2.589]	-2.864 [-3.397, -2.332]	-1.072 [-2.330, 0.187]	-1.426 [-2.212, -0.640]
	VV	-1.299 [-1.673, -0.925]	-1.214 [-1.586, -0.842]	1.279 [0.197, 2.360]	-0.002 [-0.458, 0.453]
SM	SS	1.129 [0.188, 2.070]	0.762 [-0.173, 1.696]	-2.113 [-3.152, -1.074]	-1.249 [-2.277, -0.220]
	SL	0.694 [-0.266, 1.655]	0.338 [-0.617, 1.293]	-2.527 [-3.579, -1.475]	-2.089 [-3.113, -1.066]
	SV	-3.789 [-10.991, 3.412]	-4.242 [-11.385, 2.902]	2.389 [-5.084, 9.861]	0.752 [-6.379, 7.883]
	LS	0.681 [-0.283, 1.646]	0.326 [-0.632, 1.285]	-2.689 [-3.748, -1.629]	-2.152 [-3.182, -1.122]
	LL	0.835 [-0.113, 1.783]	0.459 [-0.484, 1.402]	-2.428 [-3.475, -1.381]	-1.107 [-2.090, -0.124]
	LM	-1.803 [-2.909, -0.698]	-1.994 [-3.108, -0.880]	-1.635 [-2.841, -0.429]	-1.521 [-2.641, -0.401]
	LV	-5.394 [-7.342, -3.445]	-5.292 [-7.236, -3.349]	-0.462 [-3.006, 2.081]	-2.396 [-4.456, -0.336]
	MS	-2.538 [-5.006, -0.070]	-3.050 [-5.497, -0.603]	-2.994 [-5.410, -0.578]	-2.914 [-5.336, -0.491]
	ML	-1.955 [-3.084, -0.826]	-2.134 [-3.275, -0.993]	-1.457 [-2.727, -0.188]	-1.495 [-2.647, -0.342]
	MM	-0.742 [-1.728, 0.244]	-0.975 [-1.964, 0.015]	-1.785 [-2.880, -0.689]	-1.298 [-2.292, -0.305]
	MV	-4.458 [-5.733, -3.184]	-4.495 [-5.783, -3.207]	-4.084 [-5.716, -2.453]	-3.745 [-5.090, -2.400]
	VS	0.206 [-7.406, 7.817]	0.455 [-7.103, 8.013]	6.302 [-1.457, 14.062]	4.846 [-2.673, 12.365]
	VL	-6.448 [-8.445, -4.452]	-6.361 [-8.349, -4.373]	-2.389 [-5.020, 0.242]	-3.558 [-5.675, -1.441]

	VM	-4.236 [-5.502, -2.971]	-4.280 [-5.559, -3.001]	-3.616 [-5.232, -1.999]	-3.500 [-4.833, -2.167]
	VV	-0.962 [-2.048, 0.124]	-1.226 [-2.304, -0.147]	-0.755 [-2.166, 0.657]	-1.396 [-2.468, -0.324]
SV	SS	5.448 [-1.172, 12.067]	4.764 [-1.796, 11.325]	-6.320 [-13.319, 0.680]	-3.409 [-10.180, 3.362]
	SL	6.655 [0.029, 13.281]	5.967 [-0.605, 12.538]	-6.106 [-13.103, 0.891]	-3.623 [-10.325, 3.079]
	SM	3.789 [-3.412, 10.991]	4.242 [-2.902, 11.385]	-2.389 [-9.861, 5.084]	-0.752 [-7.883, 6.379]
	LS	6.583 [-0.043, 13.208]	5.898 [-0.673, 12.468]	-6.388 [-13.387, 0.610]	-3.720 [-10.424, 2.983]
	LL	6.205 [-0.417, 12.826]	5.575 [-0.992, 12.143]	-7.210 [-14.214, -0.205]	-3.205 [-9.949, 3.538]
	LM	0.669 [-6.011, 7.348]	0.891 [-5.743, 7.524]	-5.609 [-12.738, 1.520]	-3.200 [-9.925, 3.525]
	LV	-10.297 [-18.167, -2.427]	-9.126 [-16.936, -1.316]	-3.885 [-11.717, 3.946]	-5.888 [-13.697, 1.920]
	MS	1.505 [-5.688, 8.698]	1.480 [-5.659, 8.618]	-5.159 [-12.635, 2.317]	-3.122 [-10.254, 4.009]
	ML	0.677 [-6.010, 7.364]	0.931 [-5.710, 7.573]	-5.176 [-12.315, 1.962]	-3.233 [-9.957, 3.492]
	MM	2.692 [-3.891, 9.276]	2.904 [-3.639, 9.446]	-5.716 [-12.799, 1.368]	-2.025 [-8.831, 4.781]
	MV	-9.238 [-16.218, -2.259]	-8.292 [-15.238, -1.345]	-7.272 [-14.317, -0.227]	-8.224 [-15.163, -1.286]
	VS	6.679 [-6.632, 19.991]	7.962 [-5.225, 21.149]	6.292 [-6.736, 19.321]	6.970 [-6.077, 20.017]
	VL	-15.800 [-24.058, -7.541]	-14.403 [-22.600, -6.207]	-7.096 [-15.454, 1.261]	-9.742 [-18.009, -1.475]
	VM	-9.588 [-16.612, -2.565]	-8.608 [-15.600, -1.616]	-7.486 [-14.555, -0.417]	-8.331 [-15.315, -1.346]
	VV	-3.074 [-10.154, 4.006]	-3.066 [-10.085, 3.954]	-6.486 [-13.519, 0.548]	-4.817 [-11.808, 2.173]
LS	SS	0.220 [0.150, 0.289]	0.224 [0.155, 0.293]	0.418 [0.305, 0.532]	0.196 [0.127, 0.264]
	SL	-2.371 [-4.725, -0.018]	-2.872 [-5.206, -0.539]	-2.723 [-5.017, -0.430]	-2.743 [-5.047, -0.439]
	SM	-0.681 [-1.646, 0.283]	-0.326 [-1.285, 0.632]	2.689 [1.629, 3.748]	2.152 [1.122, 3.182]
	SV	-6.583 [-13.208, 0.043]	-5.898 [-12.468, 0.673]	6.388 [-0.610, 13.387]	3.720 [-2.983, 10.424]
	LL	0.055 [-0.025, 0.135]	0.045 [-0.035, 0.125]	0.436 [0.316, 0.556]	0.318 [0.227, 0.409]
	LM	-0.409 [-0.533, -0.285]	-0.369 [-0.499, -0.239]	0.331 [0.067, 0.594]	0.376 [0.160, 0.593]
	LV	-4.880 [-6.144, -3.615]	-4.378 [-5.643, -3.112]	1.887 [-0.192, 3.967]	0.076 [-1.521, 1.673]

	MS	-1.474 [-2.430, -0.518]	-1.276 [-2.224, -0.328]	1.374 [0.323, 2.425]	0.995 [-0.025, 2.015]
	ML	-0.396 [-0.520, -0.272]	-0.354 [-0.484, -0.224]	0.419 [0.156, 0.682]	0.424 [0.207, 0.641]
	MM	-0.187 [-0.263, -0.110]	-0.181 [-0.262, -0.099]	0.403 [0.103, 0.704]	0.127 [0.005, 0.250]
	MV	-3.211 [-3.731, -2.692]	-2.969 [-3.507, -2.432]	-1.485 [-2.746, -0.225]	-1.615 [-2.408, -0.822]
	VS	-3.399 [-10.363, 3.565]	-1.970 [-8.886, 4.945]	9.917 [2.703, 17.131]	7.454 [0.442, 14.466]
	VL	-5.573 [-6.866, -4.280]	-5.080 [-6.372, -3.789]	0.324 [-1.808, 2.457]	-0.879 [-2.514, 0.755]
	VM	-3.104 [-3.618, -2.589]	-2.866 [-3.399, -2.333]	-1.090 [-2.349, 0.169]	-1.436 [-2.222, -0.650]
	VV	-1.301 [-1.675, -0.927]	-1.216 [-1.588, -0.844]	1.261 [0.179, 2.342]	-0.008 [-0.464, 0.447]
LL	SS	0.041 [0.023, 0.060]	0.045 [0.027, 0.064]	0.168 [-0.380, 0.717]	-0.000 [-0.021, 0.021]
	SL	-0.059 [-0.139, 0.020]	-0.050 [-0.131, 0.030]	-0.440 [-0.560, -0.320]	-0.324 [-0.415, -0.234]
	SM	-0.835 [-1.783, 0.113]	-0.459 [-1.402, 0.484]	2.428 [1.381, 3.475]	1.107 [0.124, 2.090]
	SV	-6.205 [-12.826, 0.417]	-5.575 [-12.143, 0.992]	7.210 [0.205, 14.214]	3.205 [-3.538, 9.949]
	LS	-0.055 [-0.135, 0.025]	-0.045 [-0.125, 0.035]	-0.436 [-0.556, -0.316]	-0.318 [-0.409, -0.227]
	LM	-0.587 [-0.721, -0.453]	-0.520 [-0.660, -0.379]	-0.092 [-0.367, 0.184]	-0.064 [-0.266, 0.138]
	LV	-4.724 [-6.003, -3.445]	-4.225 [-5.505, -2.945]	2.305 [0.222, 4.388]	-0.749 [-2.325, 0.827]
	MS	-1.595 [-2.539, -0.651]	-1.374 [-2.311, -0.437]	1.247 [0.202, 2.293]	0.147 [-0.835, 1.129]
	ML	-0.570 [-0.703, -0.436]	-0.499 [-0.640, -0.358]	-0.014 [-0.289, 0.261]	-0.019 [-0.221, 0.183]
	MM	-0.274 [-0.334, -0.215]	-0.252 [-0.316, -0.188]	-0.852 [-1.141, -0.564]	-0.101 [-0.188, -0.013]
	MV	-3.186 [-3.710, -2.663]	-2.953 [-3.494, -2.412]	-2.564 [-3.812, -1.316]	-2.128 [-2.908, -1.347]
	VS	-2.920 [-9.876, 4.037]	-1.582 [-8.492, 5.327]	11.112 [3.894, 18.329]	7.248 [0.192, 14.303]
	VL	-5.417 [-6.723, -4.111]	-4.927 [-6.232, -3.622]	0.733 [-1.404, 2.870]	-1.670 [-3.286, -0.055]
	VM	-3.082 [-3.600, -2.563]	-2.851 [-3.388, -2.315]	-2.159 [-3.406, -0.912]	-1.950 [-2.723, -1.178]
	VV	-1.336 [-1.709, -0.963]	-1.247 [-1.618, -0.876]	0.264 [-0.834, 1.362]	-0.320 [-0.766, 0.127]
LM	SS	0.472 [0.365, 0.579]	0.414 [0.304, 0.525]	0.460 [0.134, 0.786]	0.051 [-0.120, 0.223]

	SL	0.406 [0.282, 0.530]	0.365 [0.235, 0.495]	-0.343 [-0.607, -0.080]	-0.389 [-0.606, -0.172]
	SM	1.803 [0.698, 2.909]	1.994 [0.880, 3.108]	1.635 [0.429, 2.841]	1.521 [0.401, 2.641]
	SV	-0.669 [-7.348, 6.011]	-0.891 [-7.524, 5.743]	5.609 [-1.520, 12.738]	3.200 [-3.525, 9.925]
	LS	0.409 [0.285, 0.533]	0.369 [0.239, 0.499]	-0.331 [-0.594, -0.067]	-0.376 [-0.593, -0.160]
	LL	0.587 [0.453, 0.721]	0.520 [0.379, 0.660]	0.092 [-0.184, 0.367]	0.064 [-0.138, 0.266]
	LV	-2.407 [-3.878, -0.935]	-2.321 [-3.783, -0.860]	1.370 [-0.871, 3.612]	-0.697 [-2.278, 0.884]
	MS	0.956 [-0.164, 2.077]	0.961 [-0.174, 2.096]	0.267 [-1.000, 1.534]	0.397 [-0.747, 1.541]
	ML	2.611 [0.386, 4.837]	2.902 [0.691, 5.113]	2.923 [0.726, 5.120]	2.838 [0.636, 5.039]
	MM	-0.062 [-0.280, 0.155]	-0.031 [-0.250, 0.188]	-0.196 [-0.462, 0.069]	-0.119 [-0.340, 0.103]
	MV	-2.482 [-3.159, -1.804]	-2.338 [-3.017, -1.660]	-2.285 [-3.539, -1.031]	-2.021 [-2.797, -1.246]
	VS	3.598 [-3.438, 10.634]	3.782 [-3.205, 10.769]	9.543 [2.177, 16.908]	7.298 [0.263, 14.333]
	VL	-3.208 [-4.712, -1.703]	-3.125 [-4.621, -1.629]	-0.442 [-2.766, 1.883]	-1.610 [-3.231, 0.010]
	VM	-2.346 [-3.017, -1.675]	-2.211 [-2.883, -1.539]	-1.874 [-3.126, -0.623]	-1.856 [-2.624, -1.088]
	VV	-0.621 [-1.036, -0.206]	-0.635 [-1.049, -0.221]	0.769 [-0.337, 1.875]	-0.314 [-0.758, 0.130]
LV	SS	4.558 [3.280, 5.837]	4.009 [2.731, 5.287]	-1.257 [-3.344, 0.831]	0.734 [-0.904, 2.373]
	SL	4.880 [3.615, 6.144]	4.376 [3.110, 5.642]	-1.895 [-3.974, 0.184]	-0.093 [-1.689, 1.504]
	SM	5.394 [3.445, 7.342]	5.292 [3.349, 7.236]	0.462 [-2.081, 3.006]	2.396 [0.336, 4.456]
	SV	10.297 [2.427, 18.167]	9.126 [1.316, 16.936]	3.885 [-3.946, 11.717]	5.888 [-1.920, 13.697]
	LS	4.880 [3.615, 6.144]	4.378 [3.112, 5.643]	-1.887 [-3.967, 0.192]	-0.076 [-1.673, 1.521]
	LL	4.724 [3.445, 6.003]	4.225 [2.945, 5.505]	-2.305 [-4.388, -0.222]	0.749 [-0.827, 2.325]
	LM	2.407 [0.935, 3.878]	2.321 [0.860, 3.783]	-1.370 [-3.612, 0.871]	0.697 [-0.884, 2.278]
	MS	4.131 [2.189, 6.072]	3.793 [1.851, 5.734]	-1.116 [-3.657, 1.426]	1.068 [-0.987, 3.123]
	ML	2.464 [0.983, 3.945]	2.397 [0.925, 3.868]	-1.043 [-3.317, 1.231]	0.719 [-0.865, 2.303]
	MM	2.694 [1.340, 4.047]	2.636 [1.290, 3.981]	-1.581 [-3.707, 0.546]	1.194 [-0.414, 2.802]

	MV	-4.525 [-6.824, -2.226]	-4.096 [-6.403, -1.788]	-3.871 [-6.251, -1.490]	-3.929 [-6.236, -1.623]
	VS	16.997 [8.572, 25.421]	16.418 [8.064, 24.772]	8.167 [-0.362, 16.697]	11.573 [3.171, 19.975]
	VL	-4.959 [-10.318, 0.400]	-5.112 [-10.429, 0.205]	-4.975 [-10.256, 0.305]	-4.901 [-10.192, 0.390]
	VM	-5.282 [-7.762, -2.802]	-4.793 [-7.291, -2.295]	-4.306 [-6.891, -1.721]	-4.424 [-6.925, -1.922]
	VV	2.475 [0.536, 4.413]	1.946 [0.012, 3.880]	-1.835 [-3.924, 0.254]	0.071 [-1.926, 2.067]
MS	SS	1.863 [0.924, 2.801]	1.646 [0.715, 2.576]	-0.974 [-2.012, 0.065]	-0.206 [-1.236, 0.824]
	SL	1.446 [0.487, 2.406]	1.246 [0.295, 2.197]	-1.564 [-2.622, -0.506]	-1.081 [-2.107, -0.055]
	SM	2.538 [0.070, 5.006]	3.050 [0.603, 5.497]	2.994 [0.578, 5.410]	2.914 [0.491, 5.336]
	SV	-1.505 [-8.698, 5.688]	-1.480 [-8.618, 5.659]	5.159 [-2.317, 12.635]	3.122 [-4.009, 10.254]
	LS	1.474 [0.518, 2.430]	1.276 [0.328, 2.224]	-1.374 [-2.425, -0.323]	-0.995 [-2.015, 0.025]
	LL	1.595 [0.651, 2.539]	1.374 [0.437, 2.311]	-1.247 [-2.293, -0.202]	-0.147 [-1.129, 0.835]
	LM	-0.956 [-2.077, 0.164]	-0.961 [-2.096, 0.174]	-0.267 [-1.534, 1.000]	-0.397 [-1.541, 0.747]
	LV	-4.131 [-6.072, -2.189]	-3.793 [-5.734, -1.851]	1.116 [-1.426, 3.657]	-1.068 [-3.123, 0.987]
	ML	-0.679 [-1.775, 0.417]	-0.676 [-1.784, 0.432]	-0.124 [-1.322, 1.075]	-0.254 [-1.364, 0.856]
	MM	0.094 [-0.889, 1.077]	-0.021 [-1.009, 0.968]	-0.467 [-1.558, 0.624]	-0.334 [-1.328, 0.660]
	MV	-3.412 [-4.679, -2.146]	-3.297 [-4.582, -2.012]	-2.922 [-4.543, -1.301]	-2.626 [-3.965, -1.287]
	VS	2.655 [-4.934, 10.244]	3.442 [-4.099, 10.983]	9.349 [1.599, 17.100]	7.493 [-0.016, 15.002]
	VL	-5.071 [-7.044, -3.098]	-4.747 [-6.716, -2.778]	-0.678 [-3.276, 1.920]	-2.141 [-4.234, -0.049]
	VM	-3.239 [-4.501, -1.978]	-3.130 [-4.410, -1.849]	-2.515 [-4.131, -0.898]	-2.421 [-3.753, -1.088]
	VV	-0.159 [-1.245, 0.927]	-0.263 [-1.341, 0.815]	0.225 [-1.194, 1.645]	-0.446 [-1.519, 0.627]
ML	SS	0.458 [0.352, 0.565]	0.399 [0.288, 0.510]	0.361 [0.036, 0.686]	0.018 [-0.153, 0.189]
	SL	0.393 [0.269, 0.516]	0.349 [0.220, 0.479]	-0.412 [-0.674, -0.149]	-0.430 [-0.647, -0.214]
	SM	1.955 [0.826, 3.084]	2.134 [0.993, 3.275]	1.457 [0.188, 2.727]	1.495 [0.342, 2.647]
	SV	-0.677 [-7.364, 6.010]	-0.931 [-7.573, 5.710]	5.176 [-1.962, 12.315]	3.233 [-3.492, 9.957]

	LS	0.396 [0.272, 0.520]	0.354 [0.224, 0.484]	-0.419 [-0.682, -0.156]	-0.424 [-0.641, -0.207]
	LL	0.570 [0.436, 0.703]	0.499 [0.358, 0.640]	0.014 [-0.261, 0.289]	0.019 [-0.183, 0.221]
	LM	-2.611 [-4.837, -0.386]	-2.902 [-5.113, -0.691]	-2.923 [-5.120, -0.726]	-2.838 [-5.039, -0.636]
	LV	-2.464 [-3.945, -0.983]	-2.397 [-3.868, -0.925]	1.043 [-1.231, 3.317]	-0.719 [-2.303, 0.865]
	MS	0.679 [-0.417, 1.775]	0.676 [-0.432, 1.784]	0.124 [-1.075, 1.322]	0.254 [-0.856, 1.364]
	MM	-0.097 [-0.314, 0.120]	-0.069 [-0.287, 0.149]	-0.316 [-0.580, -0.051]	-0.159 [-0.380, 0.062]
	MV	-2.536 [-3.213, -1.860]	-2.397 [-3.074, -1.720]	-2.292 [-3.544, -1.040]	-2.035 [-2.811, -1.260]
	VS	3.348 [-3.687, 10.383]	3.523 [-3.463, 10.509]	9.179 [1.828, 16.531]	7.275 [0.240, 14.310]
	VL	-3.323 [-4.822, -1.824]	-3.249 [-4.739, -1.758]	-0.649 [-2.945, 1.648]	-1.639 [-3.258, -0.020]
	VM	-2.407 [-3.081, -1.734]	-2.276 [-2.950, -1.601]	-1.931 [-3.184, -0.678]	-1.866 [-2.635, -1.097]
	VV	-0.642 [-1.057, -0.227]	-0.659 [-1.073, -0.245]	0.704 [-0.401, 1.809]	-0.312 [-0.756, 0.132]
MM	SS	0.289 [0.236, 0.341]	0.272 [0.215, 0.329]	0.479 [0.232, 0.727]	0.126 [0.023, 0.229]
	SL	0.184 [0.107, 0.261]	0.177 [0.096, 0.259]	-0.426 [-0.727, -0.126]	-0.133 [-0.256, -0.011]
	SM	0.742 [-0.244, 1.728]	0.975 [-0.015, 1.964]	1.785 [0.689, 2.880]	1.298 [0.305, 2.292]
	SV	-2.692 [-9.276, 3.891]	-2.904 [-9.446, 3.639]	5.716 [-1.368, 12.799]	2.025 [-4.781, 8.831]
	LS	0.187 [0.110, 0.263]	0.181 [0.099, 0.262]	-0.403 [-0.704, -0.103]	-0.127 [-0.250, -0.005]
	LL	0.274 [0.215, 0.334]	0.252 [0.188, 0.316]	0.852 [0.564, 1.141]	0.101 [0.013, 0.188]
	LM	0.062 [-0.155, 0.280]	0.031 [-0.188, 0.250]	0.196 [-0.069, 0.462]	0.119 [-0.103, 0.340]
	LV	-2.694 [-4.047, -1.340]	-2.636 [-3.981, -1.290]	1.581 [-0.546, 3.707]	-1.194 [-2.802, 0.414]
	MS	-0.094 [-1.077, 0.889]	0.021 [-0.968, 1.009]	0.467 [-0.624, 1.558]	0.334 [-0.660, 1.328]
	ML	0.097 [-0.120, 0.314]	0.069 [-0.149, 0.287]	0.316 [0.051, 0.580]	0.159 [-0.062, 0.380]
	MV	-2.268 [-2.949, -1.588]	-2.202 [-2.879, -1.526]	-2.027 [-3.288, -0.766]	-1.958 [-2.736, -1.180]
	VS	1.504 [-5.424, 8.432]	1.551 [-5.335, 8.437]	9.624 [2.312, 16.937]	6.324 [-0.762, 13.411]
	VL	-3.436 [-4.817, -2.055]	-3.356 [-4.730, -1.982]	-0.109 [-2.291, 2.073]	-2.184 [-3.834, -0.535]

	VM	-2.129 [-2.803, -1.456]	-2.073 [-2.742, -1.404]	-1.632 [-2.892, -0.372]	-1.794 [-2.564, -1.023]
	VV	-0.750 [-1.142, -0.359]	-0.752 [-1.142, -0.361]	1.588 [0.397, 2.779]	-0.474 [-0.930, -0.019]
MV	SS	3.135 [2.609, 3.661]	2.872 [2.330, 3.414]	2.308 [1.064, 3.553]	2.420 [1.570, 3.269]
	SL	3.211 [2.692, 3.731]	2.968 [2.431, 3.505]	1.467 [0.207, 2.727]	1.605 [0.813, 2.398]
	SM	4.458 [3.184, 5.733]	4.495 [3.207, 5.783]	4.084 [2.453, 5.716]	3.745 [2.400, 5.090]
	SV	9.238 [2.259, 16.218]	8.292 [1.345, 15.238]	7.272 [0.227, 14.317]	8.224 [1.286, 15.163]
	LS	3.211 [2.692, 3.731]	2.969 [2.432, 3.507]	1.485 [0.225, 2.746]	1.615 [0.822, 2.408]
	LL	3.186 [2.663, 3.710]	2.953 [2.412, 3.494]	2.564 [1.316, 3.812]	2.128 [1.347, 2.908]
	LM	2.482 [1.804, 3.159]	2.338 [1.660, 3.017]	2.285 [1.031, 3.539]	2.021 [1.246, 2.797]
	LV	4.525 [2.226, 6.824]	4.096 [1.788, 6.403]	3.871 [1.490, 6.251]	3.929 [1.623, 6.236]
	MS	3.412 [2.146, 4.679]	3.297 [2.012, 4.582]	2.922 [1.301, 4.543]	2.626 [1.287, 3.965]
	ML	2.536 [1.860, 3.213]	2.397 [1.720, 3.074]	2.292 [1.040, 3.544]	2.035 [1.260, 2.811]
	MM	2.268 [1.588, 2.949]	2.202 [1.526, 2.879]	2.027 [0.766, 3.288]	1.958 [1.180, 2.736]
	VS	13.152 [5.857, 20.448]	12.608 [5.359, 19.858]	11.685 [4.401, 18.968]	12.434 [5.192, 19.675]
	VL	4.058 [1.552, 6.564]	3.554 [1.021, 6.088]	3.047 [0.425, 5.669]	3.267 [0.732, 5.803]
	VM	3.941 [-1.489, 9.372]	3.677 [-1.717, 9.070]	3.696 [-1.693, 9.085]	3.654 [-1.733, 9.042]
	VV	3.447 [2.415, 4.479]	3.081 [2.018, 4.144]	2.124 [0.870, 3.377]	2.405 [1.275, 3.534]
VS	SS	2.122 [-4.831, 9.075]	0.754 [-6.146, 7.653]	-10.182 [-17.396, -2.969]	-7.471 [-14.549, -0.393]
	SL	3.378 [-3.585, 10.340]	1.951 [-4.963, 8.865]	-10.108 [-17.318, -2.897]	-7.531 [-14.542, -0.519]
	SM	-0.206 [-7.817, 7.406]	-0.455 [-8.013, 7.103]	-6.302 [-14.062, 1.457]	-4.846 [-12.365, 2.673]
	SV	-6.679 [-19.991, 6.632]	-7.962 [-21.149, 5.225]	-6.292 [-19.321, 6.736]	-6.970 [-20.017, 6.077]
	LS	3.399 [-3.565, 10.363]	1.970 [-4.945, 8.886]	-9.917 [-17.131, -2.703]	-7.454 [-14.466, -0.442]
	LL	2.920 [-4.037, 9.876]	1.582 [-5.327, 8.492]	-11.112 [-18.329, -3.894]	-7.248 [-14.303, -0.192]
	LM	-3.598 [-10.634, 3.438]	-3.782 [-10.769, 3.205]	-9.543 [-16.908, -2.177]	-7.298 [-14.333, -0.263]

	LV	-16.997 [-25.421, -8.572]	-16.418 [-24.772, -8.064]	-8.167 [-16.697, 0.362]	-11.573 [-19.975, -3.171]
	MS	-2.655 [-10.244, 4.934]	-3.442 [-10.983, 4.099]	-9.349 [-17.100, -1.599]	-7.493 [-15.002, 0.016]
	ML	-3.348 [-10.383, 3.687]	-3.523 [-10.509, 3.463]	-9.179 [-16.531, -1.828]	-7.275 [-14.310, -0.240]
	MM	-1.504 [-8.432, 5.424]	-1.551 [-8.437, 5.335]	-9.624 [-16.937, -2.312]	-6.324 [-13.411, 0.762]
	MV	-13.152 [-20.448, -5.857]	-12.608 [-19.858, -5.359]	-11.685 [-18.968, -4.401]	-12.434 [-19.675, -5.192]
	VL	-16.940 [-25.112, -8.768]	-16.587 [-24.691, -8.483]	-11.034 [-19.225, -2.843]	-13.116 [-21.240, -4.991]
	VM	-11.886 [-19.142, -4.630]	-11.421 [-18.630, -4.212]	-10.951 [-18.210, -3.692]	-11.497 [-18.695, -4.299]
	VV	-5.645 [-12.934, 1.644]	-6.432 [-13.666, 0.801]	-10.536 [-17.785, -3.288]	-8.651 [-15.859, -1.442]
VL	SS	5.248 [3.941, 6.554]	4.700 [3.396, 6.004]	0.305 [-1.835, 2.445]	1.724 [0.044, 3.405]
	SL	5.577 [4.285, 6.870]	5.083 [3.792, 6.375]	-0.286 [-2.418, 1.846]	0.873 [-0.761, 2.507]
	SM	6.448 [4.452, 8.445]	6.361 [4.373, 8.349]	2.389 [-0.242, 5.020]	3.558 [1.441, 5.675]
	SV	15.800 [7.541, 24.058]	14.403 [6.207, 22.600]	7.096 [-1.261, 15.454]	9.742 [1.475, 18.009]
	LS	5.573 [4.280, 6.866]	5.080 [3.789, 6.372]	-0.324 [-2.457, 1.808]	0.879 [-0.755, 2.514]
	LL	5.417 [4.111, 6.723]	4.927 [3.622, 6.232]	-0.733 [-2.870, 1.404]	1.670 [0.055, 3.286]
	LM	3.208 [1.703, 4.712]	3.125 [1.629, 4.621]	0.442 [-1.883, 2.766]	1.610 [-0.010, 3.231]
	LV	4.959 [-0.400, 10.318]	5.112 [-0.205, 10.429]	4.975 [-0.305, 10.256]	4.901 [-0.390, 10.192]
	MS	5.071 [3.098, 7.044]	4.747 [2.778, 6.716]	0.678 [-1.920, 3.276]	2.141 [0.049, 4.234]
	ML	3.323 [1.824, 4.822]	3.249 [1.758, 4.739]	0.649 [-1.648, 2.945]	1.639 [0.020, 3.258]
	MM	3.436 [2.055, 4.817]	3.356 [1.982, 4.730]	0.109 [-2.073, 2.291]	2.184 [0.535, 3.834]
	MV	-4.058 [-6.564, -1.552]	-3.554 [-6.088, -1.021]	-3.047 [-5.669, -0.425]	-3.267 [-5.803, -0.732]
	VS	16.940 [8.768, 25.112]	16.587 [8.483, 24.691]	11.034 [2.843, 19.225]	13.116 [4.991, 21.240]
	VM	-2.822 [-5.171, -0.472]	-2.397 [-4.766, -0.029]	-2.001 [-4.438, 0.435]	-2.258 [-4.626, 0.109]
	VV	3.635 [1.654, 5.615]	3.133 [1.161, 5.105]	-0.309 [-2.452, 1.835]	1.302 [-0.739, 3.343]
VM	SS	3.031 [2.510, 3.552]	2.772 [2.234, 3.310]	1.926 [0.683, 3.169]	2.219 [1.377, 3.060]

	SL	3.104 [2.589, 3.618]	2.864 [2.332, 3.397]	1.072 [-0.187, 2.330]	1.426 [0.640, 2.212]
	SM	4.236 [2.971, 5.502]	4.280 [3.001, 5.559]	3.616 [1.999, 5.232]	3.500 [2.167, 4.833]
	SV	9.588 [2.565, 16.612]	8.608 [1.616, 15.600]	7.486 [0.417, 14.555]	8.331 [1.346, 15.315]
	LS	3.104 [2.589, 3.618]	2.866 [2.333, 3.399]	1.090 [-0.169, 2.349]	1.436 [0.650, 2.222]
	LL	3.082 [2.563, 3.600]	2.851 [2.315, 3.388]	2.159 [0.912, 3.406]	1.950 [1.178, 2.723]
	LM	2.346 [1.675, 3.017]	2.211 [1.539, 2.883]	1.874 [0.623, 3.126]	1.856 [1.088, 2.624]
	LV	5.282 [2.802, 7.762]	4.793 [2.295, 7.291]	4.306 [1.721, 6.891]	4.424 [1.922, 6.925]
	MS	3.239 [1.978, 4.501]	3.130 [1.849, 4.410]	2.515 [0.898, 4.131]	2.421 [1.088, 3.753]
	ML	2.407 [1.734, 3.081]	2.276 [1.601, 2.950]	1.931 [0.678, 3.184]	1.866 [1.097, 2.635]
	MM	2.129 [1.456, 2.803]	2.073 [1.404, 2.742]	1.632 [0.372, 2.892]	1.794 [1.023, 2.564]
	MV	-3.941 [-9.372, 1.489]	-3.677 [-9.070, 1.717]	-3.696 [-9.085, 1.693]	-3.654 [-9.042, 1.733]
	VS	11.886 [4.630, 19.142]	11.421 [4.212, 18.630]	10.951 [3.692, 18.210]	11.497 [4.299, 18.695]
	VL	2.822 [0.472, 5.171]	2.397 [0.029, 4.766]	2.001 [-0.435, 4.438]	2.258 [-0.109, 4.626]
	VV	3.269 [2.238, 4.300]	2.905 [1.842, 3.968]	1.770 [0.518, 3.022]	2.185 [1.056, 3.313]
VV	SS	1.335 [0.963, 1.707]	1.242 [0.872, 1.611]	-0.376 [-1.467, 0.715]	0.324 [-0.151, 0.799]
	SL	1.299 [0.925, 1.673]	1.214 [0.842, 1.586]	-1.279 [-2.360, -0.197]	0.002 [-0.453, 0.458]
	SM	0.962 [-0.124, 2.048]	1.226 [0.147, 2.304]	0.755 [-0.657, 2.166]	1.396 [0.324, 2.468]
	SV	3.074 [-4.006, 10.154]	3.066 [-3.954, 10.085]	6.486 [-0.548, 13.519]	4.817 [-2.173, 11.808]
	LS	1.301 [0.927, 1.675]	1.216 [0.844, 1.588]	-1.261 [-2.342, -0.179]	0.008 [-0.447, 0.464]
	LL	1.336 [0.963, 1.709]	1.247 [0.876, 1.618]	-0.264 [-1.362, 0.834]	0.320 [-0.127, 0.766]
	LM	0.621 [0.206, 1.036]	0.635 [0.221, 1.049]	-0.769 [-1.875, 0.337]	0.314 [-0.130, 0.758]
	LV	-2.475 [-4.413, -0.536]	-1.946 [-3.880, -0.012]	1.835 [-0.254, 3.924]	-0.071 [-2.067, 1.926]
	MS	0.159 [-0.927, 1.245]	0.263 [-0.815, 1.341]	-0.225 [-1.645, 1.194]	0.446 [-0.627, 1.519]
	ML	0.642 [0.227, 1.057]	0.659 [0.245, 1.073]	-0.704 [-1.809, 0.401]	0.312 [-0.132, 0.756]

	MM	0.750 [0.359, 1.142]	0.752 [0.361, 1.142]	-1.588 [-2.779, -0.397]	0.474 [0.019, 0.930]
	MV	-3.447 [-4.479, -2.415]	-3.081 [-4.144, -2.018]	-2.124 [-3.377, -0.870]	-2.405 [-3.534, -1.275]
	VS	5.645 [-1.644, 12.934]	6.432 [-0.801, 13.666]	10.536 [3.288, 17.785]	8.651 [1.442, 15.859]
	VL	-3.635 [-5.615, -1.654]	-3.133 [-5.105, -1.161]	0.309 [-1.835, 2.452]	-1.302 [-3.343, 0.739]
	VM	-3.269 [-4.300, -2.238]	-2.905 [-3.968, -1.842]	-1.770 [-3.022, -0.518]	-2.185 [-3.313, -1.056]

BMI: body mass index; S: sedentary; L: low; M: moderate; V: vigorous; CI: confidence interval.

Swapping the comparison and baseline bigram gives equivalent estimates of association with BMI (same value but with opposite sign).

Model 1: unadjusted.
Model 2: adjusted for potential confounders (gender, exact age at age 11 clinic, parity, household social class, maternal education, maternal smoking during pregnancy and child ethnicity).
Model 3: adjusted for potential confounders (gender, exact age at age 11 clinic, parity, household social class, maternal education, maternal smoking during pregnancy and child ethnicity) and activity states (time spent in sedentary, low, moderate and vigorous activity).
Model 4: adjusted for potential confounders (gender, exact age at age 11 clinic, parity, household social class, maternal education, maternal smoking during pregnancy and child ethnicity), and mCPM.

Supplementary table 5: Difference in means of BMI for a 10 epoch pair lower frequency of baseline u-bigram, coupled with a 10 epoch pair higher frequency of comparison u-bigram

Baseline u-bigram	Comparison u-bigram	Model 1	Model 2	Model 3	Model 4
		Mean difference in BMI [95% CI] (N=4810)			
[SS]	[SL]	-0.133 [-0.172, -0.094]	-0.137 [-0.176, -0.099]	0.267 [-0.232, 0.766]	-0.105 [-0.144, -0.066]
	[SM]	-0.888 [-1.397, -0.379]	-0.720 [-1.225, -0.215]	0.774 [0.199, 1.348]	0.446 [-0.124, 1.016]
	[SV]	-2.626 [-6.570, 1.318]	-1.949 [-5.863, 1.965]	5.739 [1.492, 9.986]	3.725 [-0.358, 7.808]
	[LL]	-0.041 [-0.060, -0.023]	-0.045 [-0.064, -0.027]	-0.168 [-0.717, 0.380]	0.000 [-0.021, 0.021]
	[LM]	-0.240 [-0.293, -0.187]	-0.215 [-0.269, -0.160]	-0.411 [-0.616, -0.206]	-0.017 [-0.105, 0.072]
	[LV]	-2.609 [-3.274, -1.945]	-2.323 [-2.988, -1.659]	0.130 [-1.030, 1.289]	-0.675 [-1.551, 0.201]
	[MM]	-0.289 [-0.341, -0.236]	-0.272 [-0.329, -0.215]	-0.479 [-0.727, -0.232]	-0.126 [-0.229, -0.023]
	[MV]	-1.564 [-1.826, -1.302]	-1.437 [-1.707, -1.166]	-1.307 [-1.950, -0.664]	-1.196 [-1.627, -0.765]
	[VV]	-1.335 [-1.707, -0.963]	-1.242 [-1.611, -0.872]	0.376 [-0.715, 1.467]	-0.324 [-0.799, 0.151]
[SL]	[SM]	-0.639 [-1.159, -0.119]	-0.477 [-0.994, 0.039]	1.204 [0.620, 1.789]	0.937 [0.374, 1.501]
	[SV]	-3.459 [-7.408, 0.491]	-2.760 [-6.682, 1.162]	5.680 [1.434, 9.927]	3.774 [-0.258, 7.806]
	[LL]	0.022 [-0.025, 0.069]	0.016 [-0.031, 0.064]	0.255 [0.092, 0.418]	0.185 [0.130, 0.239]
	[LM]	-0.201 [-0.263, -0.139]	-0.180 [-0.245, -0.115]	0.188 [0.055, 0.320]	0.203 [0.095, 0.312]
	[LV]	-2.771 [-3.429, -2.113]	-2.509 [-3.167, -1.851]	0.625 [-0.524, 1.775]	-0.220 [-1.068, 0.628]
	[MM]	-0.236 [-0.295, -0.177]	-0.222 [-0.286, -0.158]	0.203 [-0.065, 0.472]	0.049 [-0.053, 0.152]
	[MV]	-1.593 [-1.853, -1.334]	-1.473 [-1.742, -1.204]	-0.687 [-1.334, -0.040]	-0.778 [-1.177, -0.379]
	[VV]	-1.352 [-1.725, -0.980]	-1.265 [-1.634, -0.895]	1.058 [-0.021, 2.136]	-0.089 [-0.538, 0.361]
[SM]	[SV]	-0.318 [-4.744, 4.109]	-0.266 [-4.665, 4.132]	4.107 [-0.553, 8.768]	2.774 [-1.619, 7.166]
	[LL]	0.704 [0.191, 1.217]	0.530 [0.020, 1.040]	-0.948 [-1.523, -0.372]	-0.376 [-0.913, 0.162]

	[LM]	-0.772 [-1.388, -0.156]	-0.839 [-1.463, -0.214]	-0.580 [-1.272, 0.112]	-0.558 [-1.186, 0.069]
	[LV]	-2.867 [-3.932, -1.803]	-2.774 [-3.837, -1.711]	-0.423 [-1.867, 1.021]	-1.337 [-2.464, -0.211]
	[MM]	-0.308 [-0.854, 0.238]	-0.406 [-0.957, 0.145]	-0.753 [-1.340, -0.167]	-0.532 [-1.084, 0.019]
	[MV]	-2.008 [-2.692, -1.323]	-2.010 [-2.704, -1.317]	-1.840 [-2.701, -0.980]	-1.671 [-2.387, -0.956]
	[VV]	-0.863 [-1.561, -0.165]	-0.946 [-1.639, -0.253]	0.322 [-0.842, 1.487]	-0.673 [-1.366, 0.019]
[SV]	[LL]	3.133 [-0.812, 7.077]	2.484 [-1.433, 6.402]	-6.389 [-10.638, -2.141]	-3.565 [-7.626, 0.496]
	[LM]	-0.804 [-4.811, 3.203]	-0.785 [-4.765, 3.195]	-5.319 [-9.666, -0.971]	-3.567 [-7.612, 0.479]
	[LV]	-10.718 [-15.718, -5.717]	-10.098 [-15.060, -5.136]	-5.642 [-10.698, -0.586]	-7.431 [-12.426, -2.436]
	[MM]	0.385 [-3.551, 4.320]	0.451 [-3.460, 4.363]	-5.538 [-9.844, -1.232]	-2.883 [-6.979, 1.214]
	[MV]	-7.071 [-11.281, -2.861]	-6.596 [-10.783, -2.409]	-6.234 [-10.502, -1.966]	-6.687 [-10.870, -2.505]
	[VV]	-3.462 [-7.783, 0.858]	-3.705 [-7.991, 0.581]	-5.671 [-9.945, -1.396]	-4.826 [-9.095, -0.558]
[LL]	[LM]	-0.301 [-0.374, -0.228]	-0.263 [-0.340, -0.186]	-0.242 [-0.409, -0.075]	-0.021 [-0.129, 0.087]
	[LV]	-2.680 [-3.347, -2.014]	-2.420 [-3.087, -1.753]	0.744 [-0.405, 1.894]	-0.658 [-1.494, 0.178]
	[MM]	-0.274 [-0.334, -0.215]	-0.252 [-0.316, -0.188]	-0.852 [-1.141, -0.564]	-0.101 [-0.188, -0.013]
	[MV]	-1.574 [-1.836, -1.311]	-1.457 [-1.729, -1.185]	-1.442 [-2.090, -0.793]	-1.036 [-1.428, -0.645]
	[VV]	-1.336 [-1.709, -0.963]	-1.247 [-1.618, -0.876]	0.264 [-0.834, 1.362]	-0.320 [-0.766, 0.127]
[LM]	[LV]	-1.547 [-2.319, -0.774]	-1.503 [-2.271, -0.735]	0.234 [-1.022, 1.489]	-0.637 [-1.476, 0.202]
	[MM]	-0.154 [-0.298, -0.010]	-0.128 [-0.274, 0.018]	-0.208 [-0.634, 0.218]	-0.123 [-0.269, 0.022]
	[MV]	-1.238 [-1.578, -0.899]	-1.169 [-1.509, -0.829]	-1.101 [-1.744, -0.457]	-0.993 [-1.383, -0.603]
	[VV]	-0.841 [-1.235, -0.447]	-0.834 [-1.225, -0.442]	0.872 [-0.215, 1.959]	-0.361 [-0.815, 0.093]
[LV]	[MM]	1.525 [0.807, 2.242]	1.496 [0.783, 2.210]	-0.546 [-1.709, 0.618]	0.869 [0.021, 1.718]
	[MV]	-2.239 [-3.524, -0.955]	-1.993 [-3.289, -0.697]	-1.836 [-3.181, -0.491]	-1.906 [-3.201, -0.611]
	[VV]	1.430 [0.209, 2.651]	1.136 [-0.081, 2.353]	0.109 [-1.165, 1.382]	0.300 [-0.937, 1.537]
[MM]	[MV]	-1.044 [-1.407, -0.682]	-1.018 [-1.378, -0.658]	-0.839 [-1.513, -0.166]	-0.908 [-1.306, -0.510]

	[VV]	-0.750 [-1.142, -0.359]	-0.752 [-1.142, -0.361]	1.588 [0.397, 2.779]	-0.474 [-0.930, -0.019]
[MV]	[VV]	1.771 [1.036, 2.505]	1.550 [0.799, 2.302]	0.075 [-1.126, 1.277]	1.278 [0.511, 2.044]

BMI: body mass index; S: sedentary; L: low; M: moderate; V: vigorous; CI: confidence interval; u-bigram: unordered bigram.

Swapping the comparison and baseline u-bigram gives equivalent estimates of association with BMI (same value but with opposite sign).

Model 1: unadjusted.
Model 2: adjusted for potential confounders (gender, exact age at age 11 clinic, parity, household social class, maternal education, maternal smoking during pregnancy and child ethnicity).
Model 3: adjusted for potential confounders (gender, exact age at age 11 clinic, parity, household social class, maternal education, maternal smoking during pregnancy and child ethnicity) and activity states (time spent in sedentary, low, moderate and vigorous activity).
Model 4: adjusted for potential confounders (gender, exact age at age 11 clinic, parity, household social class, maternal education, maternal smoking during pregnancy and child ethnicity), and mCPM.

Supplementary table 6: Number of u-bigrams equating to 1 standard deviation of our sample

U-bigram	Frequency equating to 1 standard deviation
[SS]	526.64
[SL]	225.19
[SM]	12.84
[SV]	1.66
[LL]	394.61
[LM]	123.21
[LV]	9.85
[MM]	123.04
[MV]	24.76
[VV]	17.45

Supplementary table 7: Difference in means of BMI for a 1 SD decrease of baseline u-bigram, coupled with corresponding increase in comparison u-bigram, where baseline bigram SD is less than comparison bigram SD

Baseline u-bigram	Comparison u-bigram	Model 1	Model 2	Model 3	Model 4
		Mean difference in BMI [95% CI] (N=4810)			
[SL]	[SS]	2.996 [2.123, 3.869]	3.096 [2.230, 3.963]	-6.011 [-17.254, 5.232]	2.370 [1.496, 3.245]
	[LL]	0.495 [-0.564, 1.554]	0.369 [-0.697, 1.436]	5.738 [2.066, 9.410]	4.155 [2.935, 5.375]
[SM]	[SS]	1.140 [0.487, 1.794]	0.924 [0.276, 1.573]	-0.994 [-1.732, -0.256]	-0.573 [-1.305, 0.160]
	[SL]	0.821 [0.153, 1.489]	0.613 [-0.050, 1.276]	-1.547 [-2.297, -0.796]	-1.203 [-1.927, -0.480]
	[LL]	0.904 [0.245, 1.563]	0.680 [0.025, 1.335]	-1.217 [-1.956, -0.478]	-0.482 [-1.173, 0.208]
	[LM]	-0.992 [-1.782, -0.201]	-1.077 [-1.878, -0.275]	-0.745 [-1.634, 0.144]	-0.717 [-1.523, 0.089]
	[MM]	-0.395 [-1.097, 0.306]	-0.521 [-1.229, 0.186]	-0.967 [-1.720, -0.214]	-0.683 [-1.392, 0.025]
	[MV]	-2.578 [-3.457, -1.699]	-2.582 [-3.472, -1.691]	-2.363 [-3.468, -1.258]	-2.146 [-3.065, -1.228]
	[VV]	-1.108 [-2.005, -0.212]	-1.215 [-2.105, -0.325]	0.414 [-1.081, 1.910]	-0.865 [-1.754, 0.025]
[SV]	[SS]	0.435 [-0.218, 1.087]	0.323 [-0.325, 0.970]	-0.950 [-1.653, -0.247]	-0.617 [-1.292, 0.059]
	[SL]	0.573 [-0.081, 1.226]	0.457 [-0.192, 1.106]	-0.940 [-1.643, -0.237]	-0.625 [-1.292, 0.043]
	[SM]	0.053 [-0.680, 0.785]	0.044 [-0.684, 0.772]	-0.680 [-1.451, 0.091]	-0.459 [-1.186, 0.268]
	[LL]	0.519 [-0.134, 1.171]	0.411 [-0.237, 1.060]	-1.058 [-1.761, -0.354]	-0.590 [-1.262, 0.082]
	[LM]	-0.133 [-0.796, 0.530]	-0.130 [-0.789, 0.529]	-0.880 [-1.600, -0.161]	-0.590 [-1.260, 0.079]
	[LV]	-1.774 [-2.602, -0.946]	-1.671 [-2.493, -0.850]	-0.934 [-1.771, -0.097]	-1.230 [-2.057, -0.403]
	[MM]	0.064 [-0.588, 0.715]	0.075 [-0.573, 0.722]	-0.917 [-1.629, -0.204]	-0.477 [-1.155, 0.201]
	[MV]	-1.170 [-1.867, -0.474]	-1.092 [-1.785, -0.399]	-1.032 [-1.738, -0.325]	-1.107 [-1.799, -0.415]
	[VV]	-0.573 [-1.288, 0.142]	-0.613 [-1.323, 0.096]	-0.939 [-1.646, -0.231]	-0.799 [-1.505, -0.092]
[LL]	[SS]	1.635 [0.902, 2.367]	1.786 [1.054, 2.519]	6.647 [-15.003, 28.298]	-0.005 [-0.840, 0.831]

[LM]	[SS]	2.958 [2.309, 3.607]	2.644 [1.971, 3.317]	5.063 [2.541, 7.584]	0.206 [-0.883, 1.296]
	[SL]	2.477 [1.712, 3.241]	2.220 [1.421, 3.019]	-2.314 [-3.945, -0.683]	-2.507 [-3.846, -1.168]
	[LL]	3.707 [2.803, 4.611]	3.245 [2.297, 4.193]	2.984 [0.923, 5.045]	0.260 [-1.067, 1.588]
[LV]	[SS]	2.571 [1.916, 3.226]	2.289 [1.634, 2.943]	-0.128 [-1.270, 1.014]	0.665 [-0.198, 1.528]
	[SL]	2.730 [2.081, 3.378]	2.472 [1.823, 3.120]	-0.616 [-1.748, 0.516]	0.217 [-0.618, 1.052]
	[SM]	2.825 [1.776, 3.874]	2.733 [1.686, 3.780]	0.416 [-1.006, 1.839]	1.318 [0.208, 2.427]
	[LL]	2.641 [1.984, 3.297]	2.384 [1.727, 3.041]	-0.733 [-1.866, 0.399]	0.648 [-0.176, 1.472]
	[LM]	1.524 [0.762, 2.285]	1.481 [0.724, 2.238]	-0.230 [-1.467, 1.006]	0.628 [-0.199, 1.454]
	[MM]	1.502 [0.795, 2.209]	1.474 [0.771, 2.177]	-0.538 [-1.684, 0.609]	0.857 [0.021, 1.692]
	[MV]	-2.206 [-3.472, -0.941]	-1.964 [-3.240, -0.687]	-1.809 [-3.134, -0.483]	-1.877 [-3.153, -0.602]
	[VV]	1.409 [0.206, 2.612]	1.119 [-0.080, 2.318]	0.107 [-1.148, 1.362]	0.296 [-0.923, 1.514]
[MM]	[SS]	3.551 [2.905, 4.198]	3.342 [2.641, 4.042]	5.897 [2.850, 8.945]	1.551 [0.287, 2.815]
	[SL]	2.903 [2.174, 3.632]	2.729 [1.938, 3.519]	-2.502 [-5.809, 0.805]	-0.605 [-1.865, 0.655]
	[LL]	3.377 [2.648, 4.106]	3.098 [2.310, 3.887]	10.487 [6.936, 14.037]	1.238 [0.163, 2.313]
	[LM]	1.894 [0.123, 3.665]	1.577 [-0.217, 3.371]	2.562 [-2.679, 7.803]	1.518 [-0.273, 3.309]
[MV]	[SS]	3.872 [3.224, 4.521]	3.557 [2.887, 4.226]	3.236 [1.645, 4.827]	2.961 [1.893, 4.029]
	[SL]	3.945 [3.302, 4.588]	3.647 [2.982, 4.313]	1.700 [0.098, 3.303]	1.926 [0.938, 2.913]
	[LL]	3.896 [3.245, 4.546]	3.607 [2.935, 4.280]	3.569 [1.964, 5.174]	2.565 [1.596, 3.534]
	[LM]	3.066 [2.225, 3.907]	2.895 [2.053, 3.736]	2.726 [1.133, 4.319]	2.458 [1.493, 3.423]
	[MM]	2.586 [1.688, 3.483]	2.521 [1.629, 3.413]	2.077 [0.410, 3.745]	2.247 [1.261, 3.233]
[VV]	[SS]	2.330 [1.680, 2.979]	2.167 [1.522, 2.812]	-0.656 [-2.560, 1.248]	0.566 [-0.264, 1.395]
	[SL]	2.361 [1.711, 3.010]	2.207 [1.562, 2.852]	-1.846 [-3.728, 0.036]	0.155 [-0.630, 0.939]
	[LL]	2.331 [1.680, 2.983]	2.177 [1.530, 2.824]	-0.461 [-2.378, 1.456]	0.558 [-0.221, 1.338]
	[LM]	1.468 [0.781, 2.156]	1.455 [0.771, 2.139]	-1.522 [-3.420, 0.376]	0.631 [-0.162, 1.423]

	[MM]	1.309 [0.626, 1.992]	1.312 [0.631, 1.993]	-2.772 [-4.851, -0.693]	0.828 [0.033, 1.623]
	[MV]	-3.090 [-4.372, -1.809]	-2.706 [-4.018, -1.394]	-0.131 [-2.228, 1.965]	-2.230 [-3.567, -0.892]

BMI: body mass index; S: sedentary; L: low; M: moderate; V: vigorous; CI: confidence interval; u-bigram: unordered bigram.

Results are shown where the standard deviation of the baseline u-bigram is smaller than the standard deviation of the comparison bigram, in order to determine estimates for a feasible change in activity. For example, the standard deviation of [SV] bigram is far smaller than the standard deviation change of the [SS] u-bigram, such that a 1 standard deviation = 526.65 change of [SS] would not represent a feasible change of [SV] (with standard deviation = 1.66). Hence we present the difference in means of BMI for this example for a 1 standard deviation decrease of [SV], with corresponding increase (i.e. a 1.66 increase) in frequency of the [SS] bigram.

See Supplementary table 6 for standard deviations.

Model 1: unadjusted.
Model 2: adjusted for potential confounders (gender, exact age at age 11 clinic, parity, household social class, maternal education, maternal smoking during pregnancy and child ethnicity).
Model 3: adjusted for potential confounders (gender, exact age at age 11 clinic, parity, household social class, maternal education, maternal smoking during pregnancy and child ethnicity) and activity states (time spent in sedentary, low, moderate and vigorous activity).
Model 4: adjusted for potential confounders (gender, exact age at age 11 clinic, parity, household social class, maternal education, maternal smoking during pregnancy and child ethnicity), and mCPM.

Supplementary table 8: Difference in means of BMI for a 10 epoch pair lower frequency of baseline u-bigram, coupled with a 10 epoch pair higher frequency of comparison u-bigram, for sensitivity analysis including days with at least 8 hours wear-time

Baseline u-bigram	Comparison u-bigram	Model 1	Model 2	Model 3	Model 4
		Mean difference in BMI [95% CI] (N=4810)			
[SS]	[SL]	-0.099 [-0.142, -0.056]	-0.108 [-0.151, -0.065]	-0.243 [-0.306, -0.180]	-0.123 [-0.166, -0.081]
	[SM]	-0.475 [-0.964, 0.015]	-0.342 [-0.828, 0.144]	0.609 [0.073, 1.144]	0.559 [0.020, 1.097]
	[SV]	-1.451 [-4.950, 2.048]	-0.961 [-4.433, 2.511]	4.541 [0.792, 8.290]	3.235 [-0.392, 6.861]
	[LL]	-0.019 [-0.037, -0.000]	-0.024 [-0.042, -0.005]	-0.055 [-0.161, 0.051]	0.006 [-0.014, 0.027]
	[LM]	-0.195 [-0.250, -0.140]	-0.165 [-0.222, -0.108]	-0.049 [-0.182, 0.084]	0.065 [-0.028, 0.158]
	[LV]	-2.174 [-2.802, -1.546]	-1.932 [-2.559, -1.305]	0.092 [-0.949, 1.134]	-0.718 [-1.538, 0.103]
	[MM]	-0.249 [-0.299, -0.199]	-0.230 [-0.285, -0.175]	-0.240 [-0.421, -0.058]	-0.096 [-0.196, 0.004]
	[MV]	-1.374 [-1.621, -1.126]	-1.258 [-1.514, -1.003]	-1.080 [-1.639, -0.521]	-1.236 [-1.649, -0.824]
	[VV]	-1.237 [-1.591, -0.883]	-1.148 [-1.499, -0.796]	0.296 [-0.674, 1.266]	-0.500 [-0.957, -0.043]
[SL]	[SM]	-0.534 [-1.026, -0.043]	-0.418 [-0.906, 0.069]	1.107 [0.562, 1.651]	0.877 [0.350, 1.405]
	[SV]	-2.531 [-6.061, 0.999]	-2.100 [-5.603, 1.402]	4.879 [1.132, 8.625]	3.375 [-0.201, 6.951]
	[LL]	0.014 [-0.034, 0.061]	0.016 [-0.032, 0.064]	0.244 [0.174, 0.314]	0.194 [0.139, 0.250]
	[LM]	-0.170 [-0.229, -0.112]	-0.152 [-0.213, -0.091]	0.238 [0.123, 0.353]	0.222 [0.118, 0.327]
	[LV]	-2.603 [-3.222, -1.984]	-2.385 [-3.003, -1.766]	0.656 [-0.379, 1.691]	-0.195 [-0.986, 0.597]
	[MM]	-0.199 [-0.255, -0.142]	-0.186 [-0.247, -0.125]	0.226 [0.024, 0.429]	0.085 [-0.014, 0.184]
	[MV]	-1.506 [-1.749, -1.262]	-1.396 [-1.648, -1.145]	-0.631 [-1.197, -0.065]	-0.700 [-1.076, -0.323]
	[VV]	-1.333 [-1.687, -0.979]	-1.249 [-1.600, -0.897]	0.588 [-0.379, 1.556]	-0.102 [-0.530, 0.327]
[SM]	[SV]	-0.417 [-4.389, 3.555]	-0.309 [-4.252, 3.635]	3.077 [-1.045, 7.199]	2.022 [-1.903, 5.947]

	[LL]	0.551 [0.060, 1.042]	0.435 [-0.052, 0.923]	-0.678 [-1.212, -0.144]	-0.701 [-1.229, -0.172]
	[LM]	-0.705 [-1.279, -0.131]	-0.751 [-1.331, -0.171]	-0.508 [-1.139, 0.124]	-0.493 [-1.074, 0.088]
	[LV]	-2.912 [-3.913, -1.911]	-2.783 [-3.782, -1.784]	-0.670 [-1.992, 0.652]	-1.449 [-2.501, -0.397]
	[MM]	-0.360 [-0.880, 0.159]	-0.416 [-0.939, 0.106]	-0.775 [-1.308, -0.242]	-0.653 [-1.179, -0.126]
	[MV]	-2.061 [-2.710, -1.412]	-2.021 [-2.677, -1.364]	-1.735 [-2.519, -0.950]	-1.713 [-2.386, -1.040]
	[VV]	-1.088 [-1.755, -0.420]	-1.110 [-1.772, -0.448]	-0.153 [-1.213, 0.908]	-0.907 [-1.567, -0.248]
[SV]	[LL]	2.274 [-1.236, 5.784]	1.818 [-1.667, 5.303]	-4.969 [-8.718, -1.220]	-3.962 [-7.582, -0.343]
	[LM]	-0.914 [-4.478, 2.649]	-0.880 [-4.417, 2.657]	-4.237 [-8.050, -0.424]	-3.666 [-7.256, -0.076]
	[LV]	-9.598 [-14.011, -5.186]	-9.058 [-13.435, -4.681]	-4.936 [-9.399, -0.473]	-6.562 [-10.973, -2.152]
	[MM]	-0.046 [-3.547, 3.456]	0.077 [-3.401, 3.556]	-4.930 [-8.691, -1.169]	-3.267 [-6.916, 0.383]
	[MV]	-6.617 [-10.355, -2.879]	-6.146 [-9.863, -2.429]	-5.523 [-9.292, -1.754]	-6.140 [-9.852, -2.427]
	[VV]	-3.730 [-7.562, 0.101]	-3.839 [-7.640, -0.038]	-4.636 [-8.422, -0.849]	-4.726 [-8.510, -0.943]
[LL]	[LM]	-0.272 [-0.341, -0.203]	-0.236 [-0.308, -0.164]	-0.025 [-0.164, 0.115]	0.048 [-0.058, 0.153]
	[LV]	-2.487 [-3.108, -1.866]	-2.256 [-2.878, -1.635]	0.190 [-0.852, 1.231]	-0.441 [-1.234, 0.352]
	[MM]	-0.251 [-0.307, -0.195]	-0.229 [-0.289, -0.169]	-0.391 [-0.606, -0.177]	-0.050 [-0.137, 0.037]
	[MV]	-1.460 [-1.705, -1.215]	-1.356 [-1.609, -1.102]	-1.182 [-1.750, -0.614]	-0.901 [-1.285, -0.516]
	[VV]	-1.311 [-1.666, -0.957]	-1.224 [-1.576, -0.871]	0.229 [-0.748, 1.205]	-0.276 [-0.706, 0.155]
[LM]	[LV]	-1.520 [-2.242, -0.797]	-1.465 [-2.183, -0.748]	-0.103 [-1.241, 1.035]	-0.504 [-1.290, 0.283]
	[MM]	-0.155 [-0.292, -0.018]	-0.122 [-0.261, 0.016]	-0.123 [-0.301, 0.055]	-0.149 [-0.287, -0.010]
	[MV]	-1.205 [-1.525, -0.885]	-1.123 [-1.443, -0.803]	-1.034 [-1.601, -0.468]	-0.899 [-1.270, -0.528]
	[VV]	-0.877 [-1.252, -0.502]	-0.855 [-1.228, -0.483]	0.362 [-0.613, 1.338]	-0.250 [-0.700, 0.200]
[LV]	[MM]	1.448 [0.779, 2.117]	1.430 [0.765, 2.095]	-0.481 [-1.516, 0.555]	0.705 [-0.112, 1.522]
	[MV]	-2.095 [-3.292, -0.898]	-1.842 [-3.048, -0.635]	-1.645 [-2.892, -0.398]	-1.741 [-2.947, -0.534]
	[VV]	1.071 [-0.067, 2.210]	0.856 [-0.278, 1.990]	-0.010 [-1.195, 1.174]	0.054 [-1.100, 1.208]

[MM]	[MV]	-1.013 [-1.351, -0.674]	-0.980 [-1.316, -0.643]	-0.866 [-1.477, -0.255]	-0.857 [-1.231, -0.483]
	[VV]	-0.785 [-1.157, -0.413]	-0.778 [-1.148, -0.408]	0.683 [-0.360, 1.725]	-0.473 [-0.918, -0.029]
[MV]	[VV]	1.508 [0.815, 2.202]	1.322 [0.612, 2.032]	0.367 [-0.605, 1.339]	1.046 [0.320, 1.771]

Results for sensitivity analysis, including only days with at least 8 hours wear time in analyses.

BMI: body mass index; S: sedentary; L: low; M: moderate; V: vigorous; CI: confidence interval; u-bigram: unordered bigram.

Swapping the comparison and baseline u-bigram gives equivalent estimates of association with BMI (same value but with opposite sign).

Model 1: unadjusted.
Model 2: adjusted for potential confounders (gender, exact age at age 11 clinic, parity, household social class, maternal education, maternal smoking during pregnancy and child ethnicity).
Model 3: adjusted for potential confounders (gender, exact age at age 11 clinic, parity, household social class, maternal education, maternal smoking during pregnancy and child ethnicity) and activity states (time spent in sedentary, low, moderate and vigorous activity).
Model 4: adjusted for potential confounders (gender, exact age at age 11 clinic, parity, household social class, maternal education, maternal smoking during pregnancy and child ethnicity), and mCPM.

Supplementary figure 1: Distributions of the average number of occurrences of bigrams per day

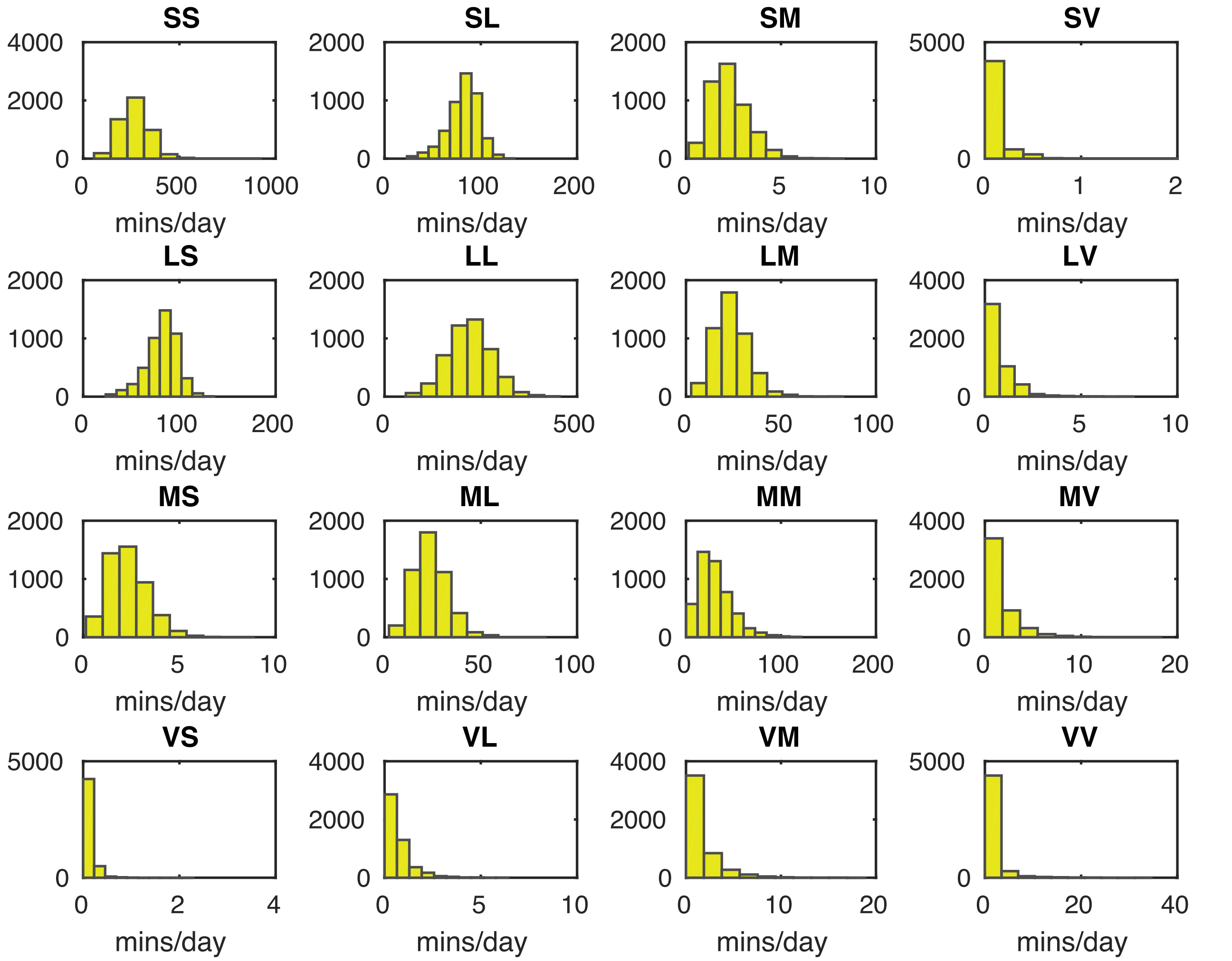

Supplementary figure 2: Difference in means of BMI for a 10 epoch pair lower frequency of SS bigram, coupled with a 10 epoch pair higher frequency of comparison bigram

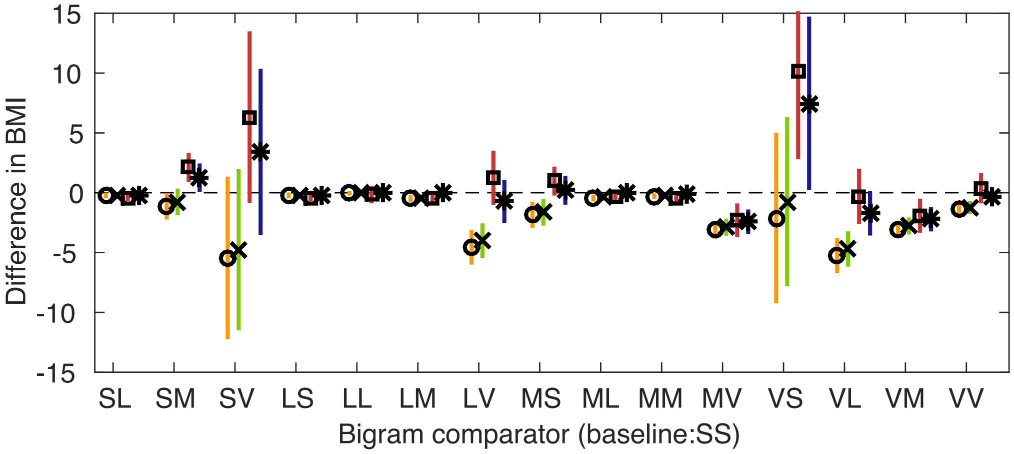

Swapping the comparison and baseline bigram gives equivalent estimates of association with BMI (same value but with opposite sign).

Model 1 (circle): unadjusted.
Model 2 (cross): adjusted for potential confounders (gender, exact age at age 11 clinic, parity, household social class, maternal education, maternal smoking during pregnancy and child ethnicity).
Model 3 (square): adjusted for potential confounders (gender, exact age at age 11 clinic, parity, household social class, maternal education, maternal smoking during pregnancy and child ethnicity) and activity states (time spent in sedentary, low, moderate and vigorous activity).
Model 4 (star): adjusted for potential confounders (gender, exact age at age 11 clinic, parity, household social class, maternal education, maternal smoking during pregnancy and child ethnicity), and mCPM.

Supplementary figure 3: Difference in means of BMI for a 10 epoch pair lower frequency of SL bigram, coupled with a 10 epoch pair higher frequency of comparison bigram

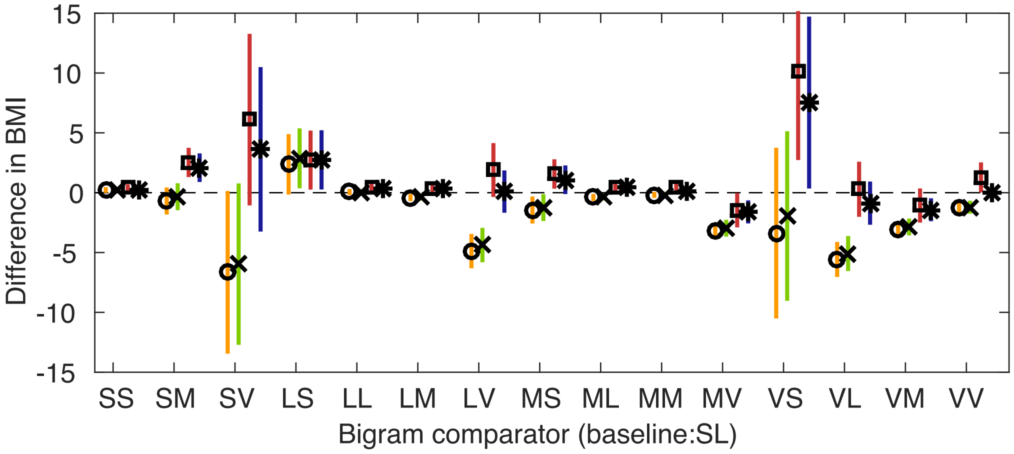

Swapping the comparison and baseline bigram gives equivalent estimates of association with BMI (same value but with opposite sign).

Model 1 (circle): unadjusted.
Model 2 (cross): adjusted for potential confounders (gender, exact age at age 11 clinic, parity, household social class, maternal education, maternal smoking during pregnancy and child ethnicity).
Model 3 (square): adjusted for potential confounders (gender, exact age at age 11 clinic, parity, household social class, maternal education, maternal smoking during pregnancy and child ethnicity) and activity states (time spent in sedentary, low, moderate and vigorous activity).
Model 4 (star): adjusted for potential confounders (gender, exact age at age 11 clinic, parity, household social class, maternal education, maternal smoking during pregnancy and child ethnicity), and mCPM.

Supplementary figure 4: Difference in means of BMI for a 10 epoch pair lower frequency of SM bigram, coupled with a 10 epoch pair higher frequency of comparison bigram

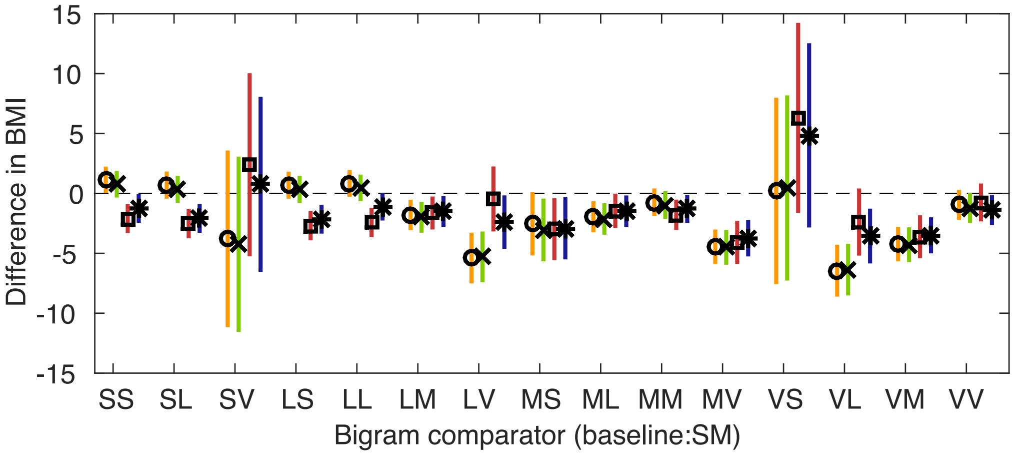

Swapping the comparison and baseline bigram gives equivalent estimates of association with BMI (same value but with opposite sign).

Model 1 (circle): unadjusted.
Model 2 (cross): adjusted for potential confounders (gender, exact age at age 11 clinic, parity, household social class, maternal education, maternal smoking during pregnancy and child ethnicity).
Model 3 (square): adjusted for potential confounders (gender, exact age at age 11 clinic, parity, household social class, maternal education, maternal smoking during pregnancy and child ethnicity) and activity states (time spent in sedentary, low, moderate and vigorous activity).
Model 4 (star): adjusted for potential confounders (gender, exact age at age 11 clinic, parity, household social class, maternal education, maternal smoking during pregnancy and child ethnicity), and mCPM.

Supplementary figure 5: Difference in means of BMI for a 10 epoch pair lower frequency of SV bigram, coupled with a 10 epoch pair higher frequency of comparison bigram

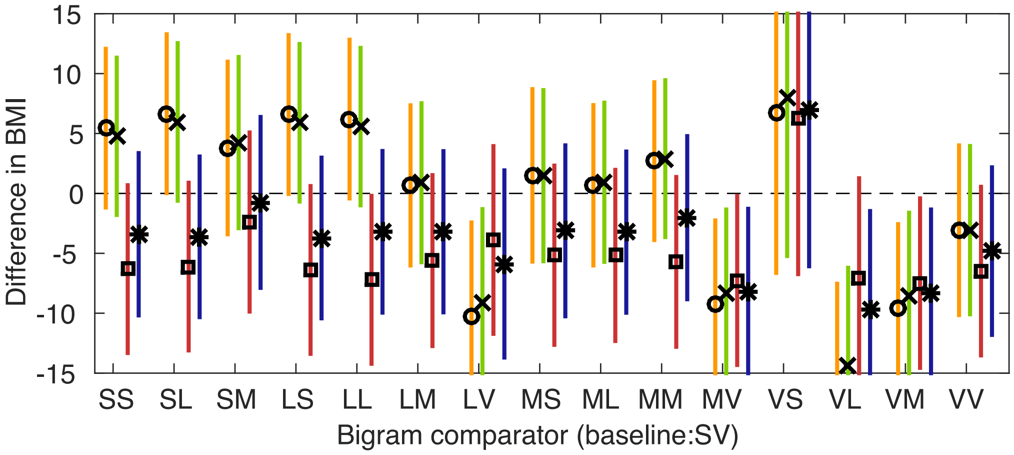

Swapping the comparison and baseline bigram gives equivalent estimates of association with BMI (same value but with opposite sign).

Model 1 (circle): unadjusted.
Model 2 (cross): adjusted for potential confounders (gender, exact age at age 11 clinic, parity, household social class, maternal education, maternal smoking during pregnancy and child ethnicity).
Model 3 (square): adjusted for potential confounders (gender, exact age at age 11 clinic, parity, household social class, maternal education, maternal smoking during pregnancy and child ethnicity) and activity states (time spent in sedentary, low, moderate and vigorous activity).
Model 4 (star): adjusted for potential confounders (gender, exact age at age 11 clinic, parity, household social class, maternal education, maternal smoking during pregnancy and child ethnicity), and mCPM.

Supplementary figure 6: Difference in means of BMI for a 10 epoch pair lower frequency of LS bigram, coupled with a 10 epoch pair higher frequency of comparison bigram

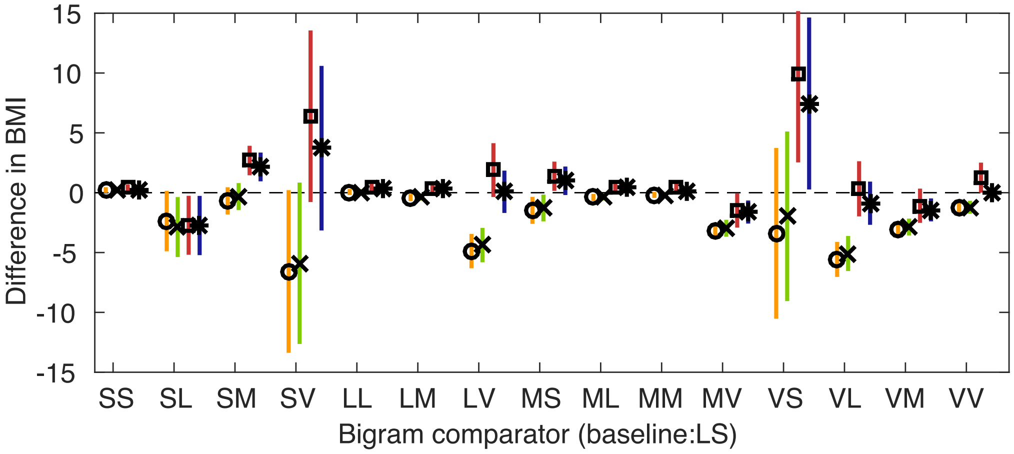

Swapping the comparison and baseline bigram gives equivalent estimates of association with BMI (same value but with opposite sign).

Model 1 (circle): unadjusted.
Model 2 (cross): adjusted for potential confounders (gender, exact age at age 11 clinic, parity, household social class, maternal education, maternal smoking during pregnancy and child ethnicity).
Model 3 (square): adjusted for potential confounders (gender, exact age at age 11 clinic, parity, household social class, maternal education, maternal smoking during pregnancy and child ethnicity) and activity states (time spent in sedentary, low, moderate and vigorous activity).
Model 4 (star): adjusted for potential confounders (gender, exact age at age 11 clinic, parity, household social class, maternal education, maternal smoking during pregnancy and child ethnicity), and mCPM.

Supplementary figure 7: Difference in means of BMI for a 10 epoch pair lower frequency of LL bigram, coupled with a 10 epoch pair higher frequency of comparison bigram

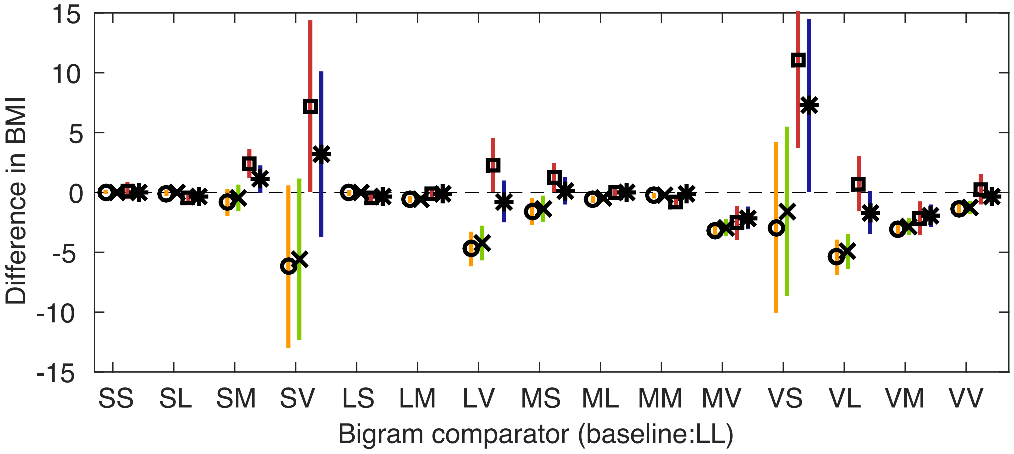

Swapping the comparison and baseline bigram gives equivalent estimates of association with BMI (same value but with opposite sign).

Model 1 (circle): unadjusted.
Model 2 (cross): adjusted for potential confounders (gender, exact age at age 11 clinic, parity, household social class, maternal education, maternal smoking during pregnancy and child ethnicity).
Model 3 (square): adjusted for potential confounders (gender, exact age at age 11 clinic, parity, household social class, maternal education, maternal smoking during pregnancy and child ethnicity) and activity states (time spent in sedentary, low, moderate and vigorous activity).
Model 4 (star): adjusted for potential confounders (gender, exact age at age 11 clinic, parity, household social class, maternal education, maternal smoking during pregnancy and child ethnicity), and mCPM.

Supplementary figure 8: Difference in means of BMI for a 10 epoch pair lower frequency of LM bigram, coupled with a 10 epoch pair higher frequency of comparison bigram

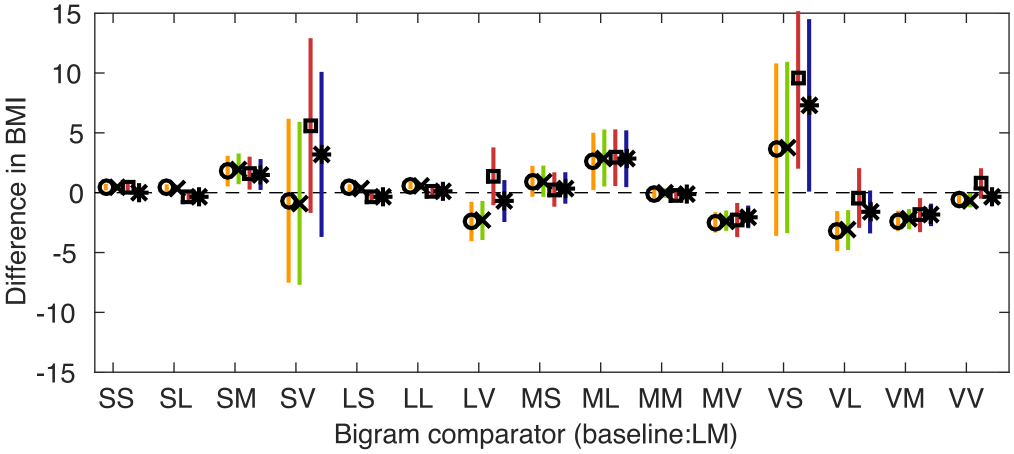

Swapping the comparison and baseline bigram gives equivalent estimates of association with BMI (same value but with opposite sign).

Model 1 (circle): unadjusted.
Model 2 (cross): adjusted for potential confounders (gender, exact age at age 11 clinic, parity, household social class, maternal education, maternal smoking during pregnancy and child ethnicity).
Model 3 (square): adjusted for potential confounders (gender, exact age at age 11 clinic, parity, household social class, maternal education, maternal smoking during pregnancy and child ethnicity) and activity states (time spent in sedentary, low, moderate and vigorous activity).
Model 4 (star): adjusted for potential confounders (gender, exact age at age 11 clinic, parity, household social class, maternal education, maternal smoking during pregnancy and child ethnicity), and mCPM.

Supplementary figure 9: Difference in means of BMI for a 10 epoch pair lower frequency of LV bigram, coupled with a 10 epoch pair higher frequency of comparison bigram

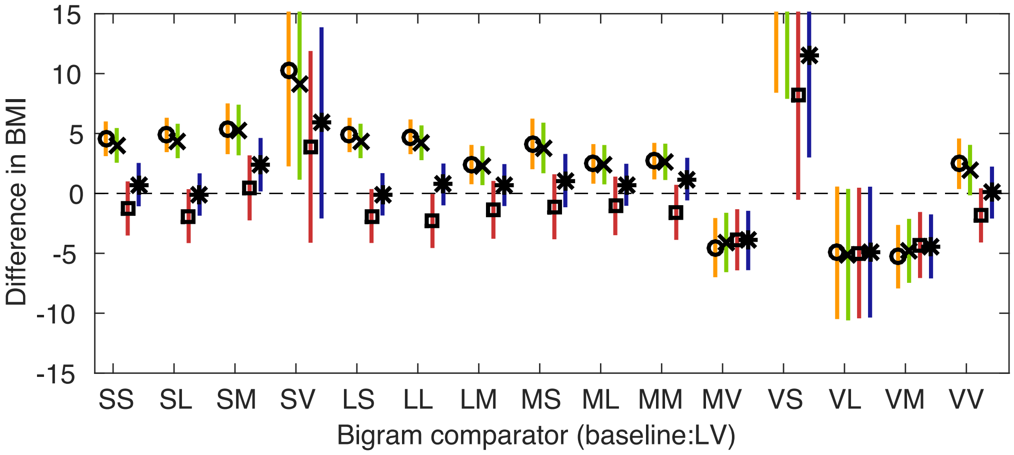

Swapping the comparison and baseline bigram gives equivalent estimates of association with BMI (same value but with opposite sign).

Model 1 (circle): unadjusted.
Model 2 (cross): adjusted for potential confounders (gender, exact age at age 11 clinic, parity, household social class, maternal education, maternal smoking during pregnancy and child ethnicity).
Model 3 (square): adjusted for potential confounders (gender, exact age at age 11 clinic, parity, household social class, maternal education, maternal smoking during pregnancy and child ethnicity) and activity states (time spent in sedentary, low, moderate and vigorous activity).
Model 4 (star): adjusted for potential confounders (gender, exact age at age 11 clinic, parity, household social class, maternal education, maternal smoking during pregnancy and child ethnicity), and mCPM.

Supplementary figure 10: Difference in means of BMI for a 10 epoch pair lower frequency of MS bigram, coupled with a 10 epoch pair higher frequency of comparison bigram

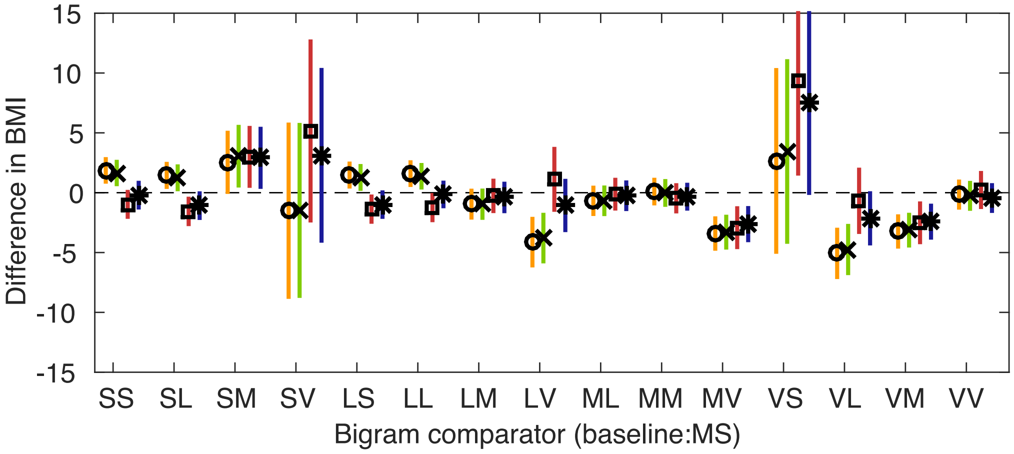

Swapping the comparison and baseline bigram gives equivalent estimates of association with BMI (same value but with opposite sign).

Model 1 (circle): unadjusted.
Model 2 (cross): adjusted for potential confounders (gender, exact age at age 11 clinic, parity, household social class, maternal education, maternal smoking during pregnancy and child ethnicity).
Model 3 (square): adjusted for potential confounders (gender, exact age at age 11 clinic, parity, household social class, maternal education, maternal smoking during pregnancy and child ethnicity) and activity states (time spent in sedentary, low, moderate and vigorous activity).
Model 4 (star): adjusted for potential confounders (gender, exact age at age 11 clinic, parity, household social class, maternal education, maternal smoking during pregnancy and child ethnicity), and mCPM.

Supplementary figure 11: Difference in means of BMI for a 10 epoch pair lower frequency of ML bigram, coupled with a 10 epoch pair higher frequency of comparison bigram

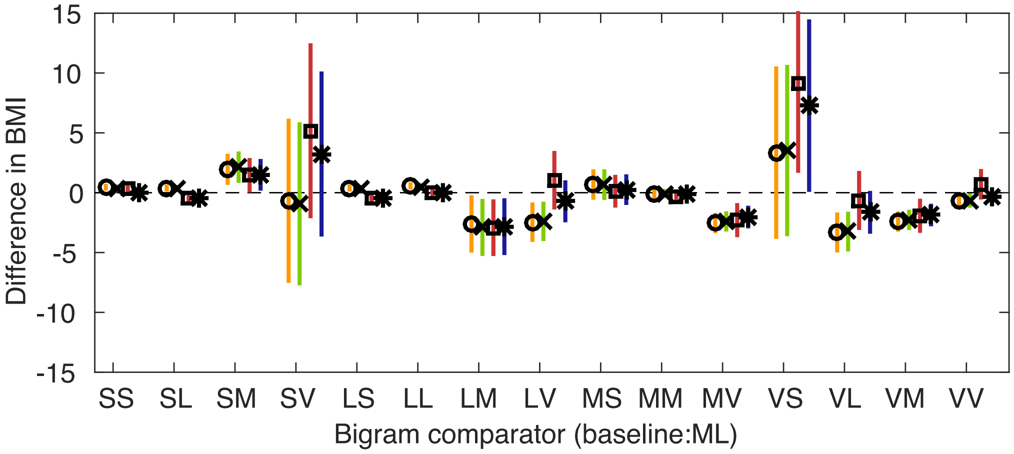

Swapping the comparison and baseline bigram gives equivalent estimates of association with BMI (same value but with opposite sign).

Model 1 (circle): unadjusted.
Model 2 (cross): adjusted for potential confounders (gender, exact age at age 11 clinic, parity, household social class, maternal education, maternal smoking during pregnancy and child ethnicity).
Model 3 (square): adjusted for potential confounders (gender, exact age at age 11 clinic, parity, household social class, maternal education, maternal smoking during pregnancy and child ethnicity) and activity states (time spent in sedentary, low, moderate and vigorous activity).
Model 4 (star): adjusted for potential confounders (gender, exact age at age 11 clinic, parity, household social class, maternal education, maternal smoking during pregnancy and child ethnicity), and mCPM.

Supplementary figure 12: Difference in means of BMI for a 10 epoch pair lower frequency of MM bigram, coupled with a 10 epoch pair higher frequency of comparison bigram

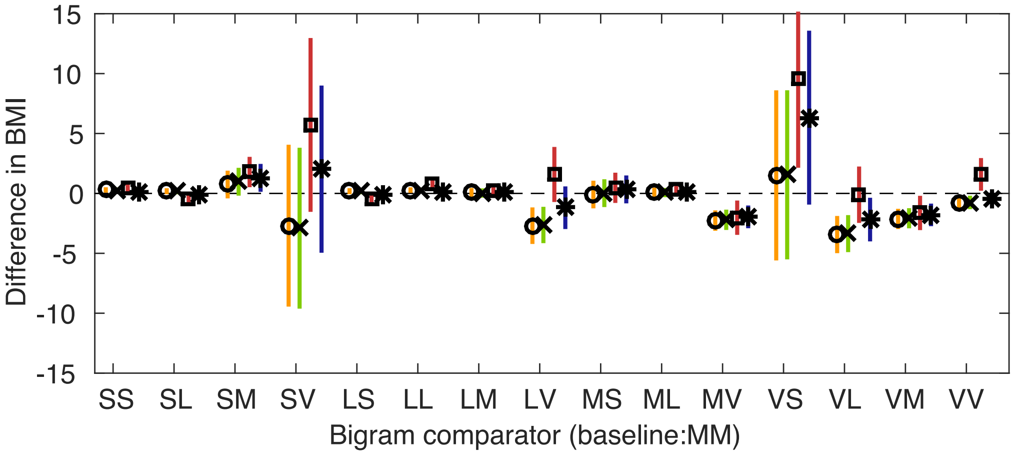

Swapping the comparison and baseline bigram gives equivalent estimates of association with BMI (same value but with opposite sign).

Model 1 (circle): unadjusted.
Model 2 (cross): adjusted for potential confounders (gender, exact age at age 11 clinic, parity, household social class, maternal education, maternal smoking during pregnancy and child ethnicity).
Model 3 (square): adjusted for potential confounders (gender, exact age at age 11 clinic, parity, household social class, maternal education, maternal smoking during pregnancy and child ethnicity) and activity states (time spent in sedentary, low, moderate and vigorous activity).
Model 4 (star): adjusted for potential confounders (gender, exact age at age 11 clinic, parity, household social class, maternal education, maternal smoking during pregnancy and child ethnicity), and mCPM.

Supplementary figure 13: Difference in means of BMI for a 10 epoch pair lower frequency of MV bigram, coupled with a 10 epoch pair higher frequency of comparison bigram

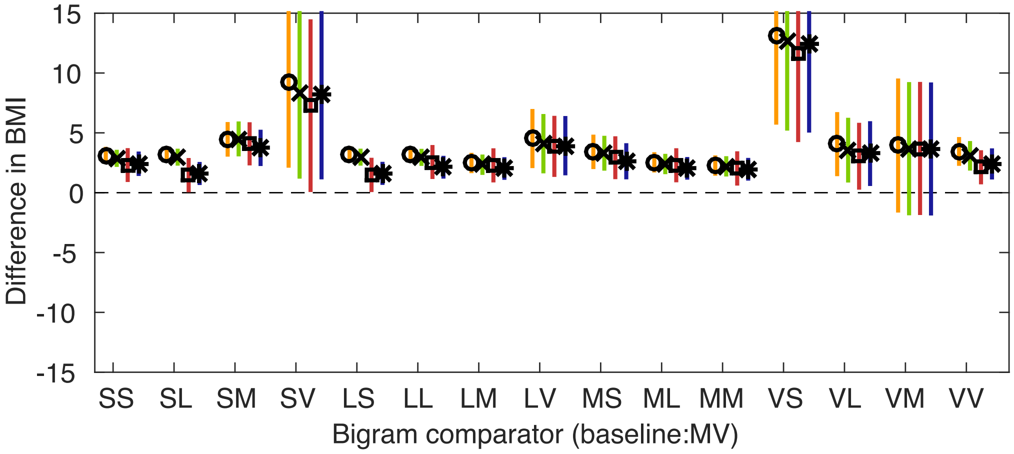

Swapping the comparison and baseline bigram gives equivalent estimates of association with BMI (same value but with opposite sign).

Model 1 (circle): unadjusted.
Model 2 (cross): adjusted for potential confounders (gender, exact age at age 11 clinic, parity, household social class, maternal education, maternal smoking during pregnancy and child ethnicity).
Model 3 (square): adjusted for potential confounders (gender, exact age at age 11 clinic, parity, household social class, maternal education, maternal smoking during pregnancy and child ethnicity) and activity states (time spent in sedentary, low, moderate and vigorous activity).
Model 4 (star): adjusted for potential confounders (gender, exact age at age 11 clinic, parity, household social class, maternal education, maternal smoking during pregnancy and child ethnicity), and mCPM.

Supplementary figure 14: Difference in means of BMI for a 10 epoch pair lower frequency of VS bigram, coupled with a 10 epoch pair higher frequency of comparison bigram

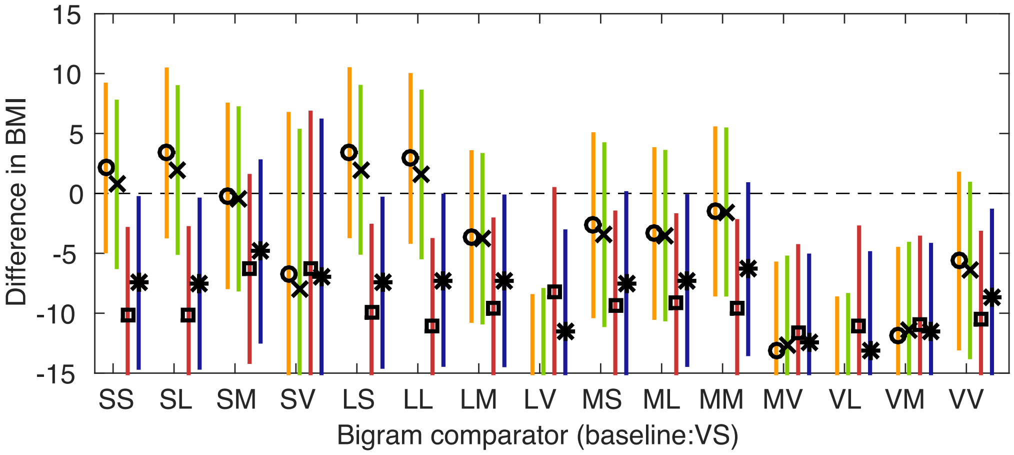

Swapping the comparison and baseline bigram gives equivalent estimates of association with BMI (same value but with opposite sign).

Model 1 (circle): unadjusted.
Model 2 (cross): adjusted for potential confounders (gender, exact age at age 11 clinic, parity, household social class, maternal education, maternal smoking during pregnancy and child ethnicity).
Model 3 (square): adjusted for potential confounders (gender, exact age at age 11 clinic, parity, household social class, maternal education, maternal smoking during pregnancy and child ethnicity) and activity states (time spent in sedentary, low, moderate and vigorous activity).
Model 4 (star): adjusted for potential confounders (gender, exact age at age 11 clinic, parity, household social class, maternal education, maternal smoking during pregnancy and child ethnicity), and mCPM.

Supplementary figure 15: Difference in means of BMI for a 10 epoch pair lower frequency of VL bigram, coupled with a 10 epoch pair higher frequency of comparison bigram

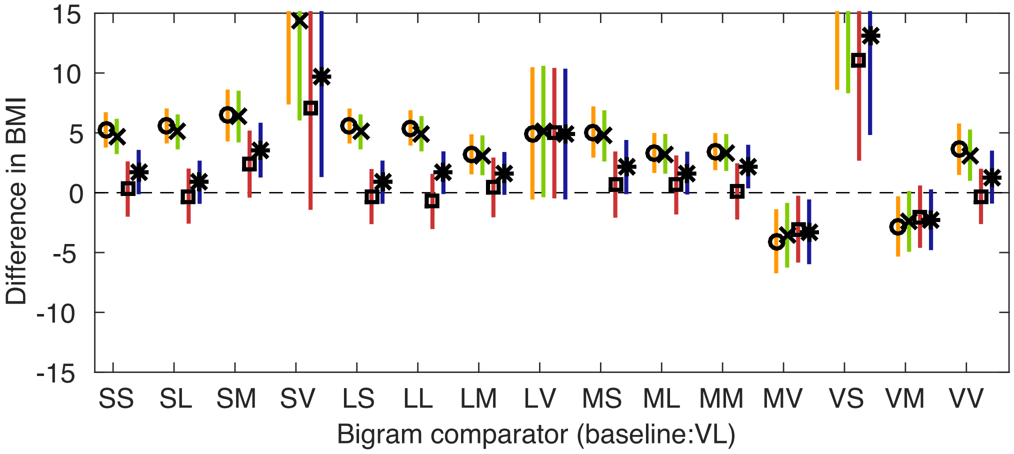

Swapping the comparison and baseline bigram gives equivalent estimates of association with BMI (same value but with opposite sign).

Model 1 (circle): unadjusted.
Model 2 (cross): adjusted for potential confounders (gender, exact age at age 11 clinic, parity, household social class, maternal education, maternal smoking during pregnancy and child ethnicity).
Model 3 (square): adjusted for potential confounders (gender, exact age at age 11 clinic, parity, household social class, maternal education, maternal smoking during pregnancy and child ethnicity) and activity states (time spent in sedentary, low, moderate and vigorous activity).
Model 4 (star): adjusted for potential confounders (gender, exact age at age 11 clinic, parity, household social class, maternal education, maternal smoking during pregnancy and child ethnicity), and mCPM.

Supplementary figure 16: Difference in means of BMI for a 10 epoch pair lower frequency of VM bigram, coupled with a 10 epoch pair higher frequency of comparison bigram

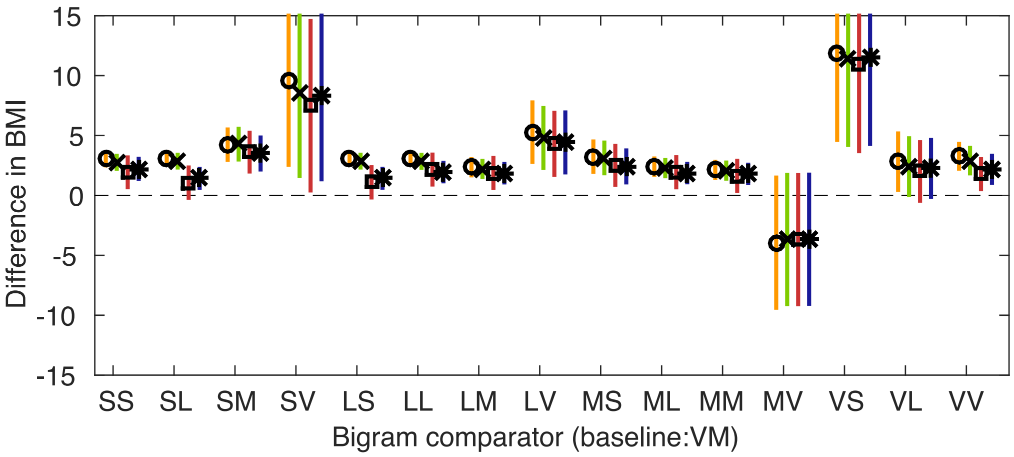

Swapping the comparison and baseline bigram gives equivalent estimates of association with BMI (same value but with opposite sign).

Model 1 (circle): unadjusted.
Model 2 (cross): adjusted for potential confounders (gender, exact age at age 11 clinic, parity, household social class, maternal education, maternal smoking during pregnancy and child ethnicity).
Model 3 (square): adjusted for potential confounders (gender, exact age at age 11 clinic, parity, household social class, maternal education, maternal smoking during pregnancy and child ethnicity) and activity states (time spent in sedentary, low, moderate and vigorous activity).
Model 4 (star): adjusted for potential confounders (gender, exact age at age 11 clinic, parity, household social class, maternal education, maternal smoking during pregnancy and child ethnicity), and mCPM.

Supplementary figure 17: Difference in means of BMI for a 10 epoch pair lower frequency of VV bigram, coupled with a 10 epoch pair higher frequency of comparison bigram

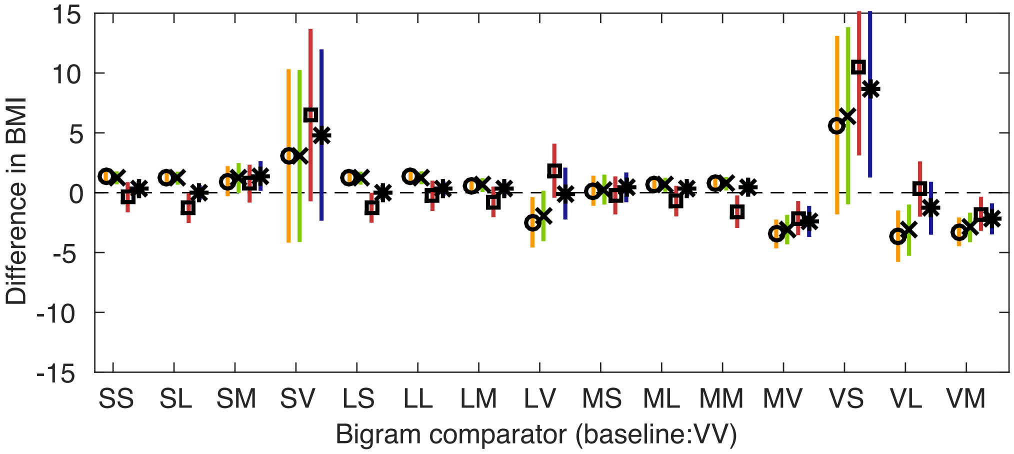

Swapping the comparison and baseline u-bigram gives equivalent estimates of association with BMI (same value but with opposite sign).

Model 1 (circle): unadjusted.
Model 2 (cross): adjusted for potential confounders (gender, exact age at age 11 clinic, parity, household social class, maternal education, maternal smoking during pregnancy and child ethnicity).
Model 3 (square): adjusted for potential confounders (gender, exact age at age 11 clinic, parity, household social class, maternal education, maternal smoking during pregnancy and child ethnicity) and activity states (time spent in sedentary, low, moderate and vigorous activity).
Model 4 (star): adjusted for potential confounders (gender, exact age at age 11 clinic, parity, household social class, maternal education, maternal smoking during pregnancy and child ethnicity), and mCPM.

Supplementary figure 18: Difference in means of BMI for 1 standard deviation decrease of baseline u-bigram, coupled with an equivalent increase of comparison u-bigram, where baseline bigram standard deviation is less than comparison bigram standard deviation

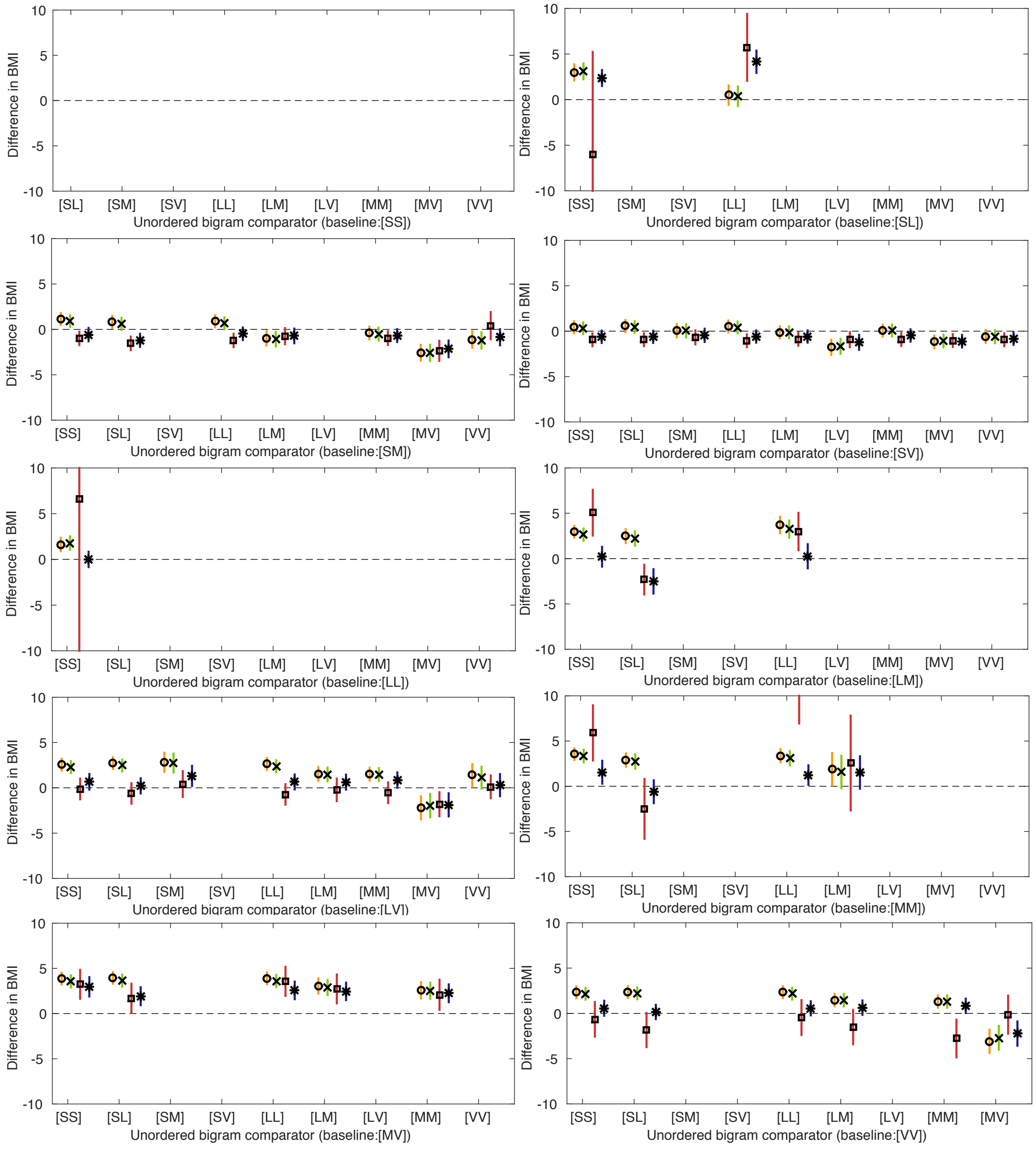

Results are shown where the standard deviation of the baseline u-bigram is smaller than the standard deviation of the comparison bigram, in order to determine estimates for a feasible change in activity. For example, the standard deviation of [SV] bigram is far smaller than the standard deviation change of the [SS] u-bigram, such that a 1 standard deviation = 526.65 change of [SS] would not represent a feasible change of [SV] (with standard deviation = 1.66). Hence we present the difference in means of BMI for this example for a 1 standard deviation decrease of [SV], with corresponding increase (i.e. a 1.66 increase) in frequency of the [SS] bigram.

BMI: body mass index; u-bigram: unordered-bigram; S: sedentary; L: low; M: moderate; V: vigorous.

Model 1 (circle): unadjusted.

Model 2 (cross): adjusted for potential confounders (gender, exact age at age 11 clinic, parity, household social class, maternal education, maternal smoking during pregnancy and child ethnicity).
Model 3 (square): adjusted for potential confounders (gender, exact age at age 11 clinic, parity, household social class, maternal education, maternal smoking during pregnancy and child ethnicity) and activity states (time spent in sedentary, low, moderate and vigorous activity).
Model 4 (star): adjusted for potential confounders (gender, exact age at age 11 clinic, parity, household social class, maternal education, maternal smoking during pregnancy and child ethnicity), and mCPM.
N=4810

Supplementary figure 19: Difference in means of BMI for a 10 epoch pair lower frequency of baseline activity u-bigram, coupled with a 10 epoch pair higher frequency of comparison activity u-bigram, for sensitivity analysis including days with at least 8 hours wear-time

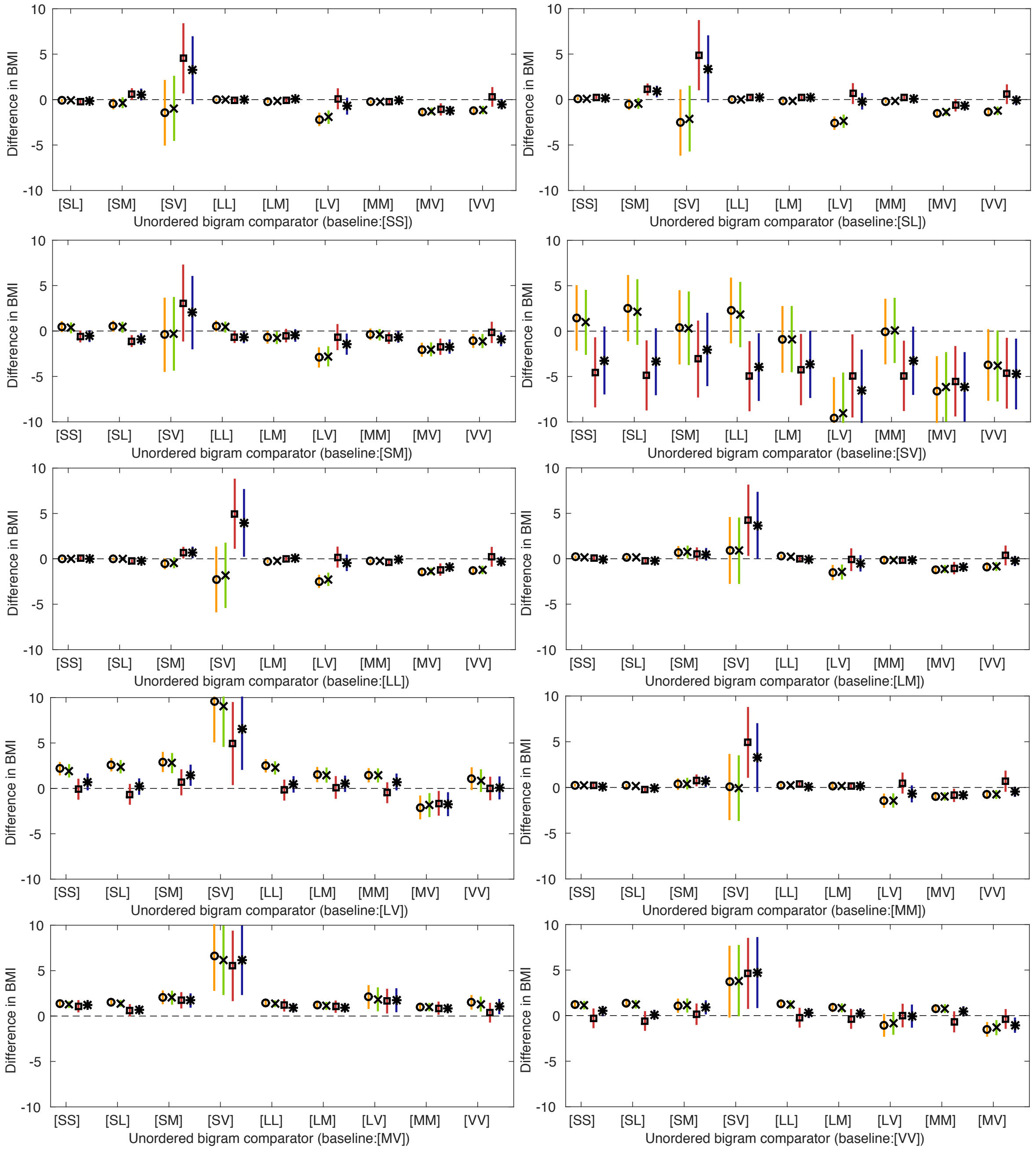

Results for sensitivity analysis, including only days with at least 8 hours wear time in analyses.

BMI: body mass index; u-bigram: unordered-bigram; S: sedentary; L: low; M: moderate; V: vigorous.

Swapping the comparison and baseline u-bigram gives equivalent estimates of association with BMI (same values but with opposite sign).

Model 1 (circle): unadjusted.
Model 2 (cross): adjusted for potential confounders (gender, exact age at age 11 clinic, parity, household social class, maternal education, maternal smoking during pregnancy and child ethnicity).

Model 3 (square): adjusted for potential confounders (gender, exact age at age 11 clinic, parity, household social class, maternal education, maternal smoking during pregnancy and child ethnicity) and activity states (time spent in sedentary, low, moderate and vigorous activity).
Model 4 (star): adjusted for potential confounders (gender, exact age at age 11 clinic, parity, household social class, maternal education, maternal smoking during pregnancy and child ethnicity), and mCPM.
N=4810
